# Neutrophil extracellular traps mediate cardiomyocyte ferroptosis via the Hippo–Yap pathway to exacerbate doxorubicin-induced cardiotoxicity

**DOI:** 10.1007/s00018-024-05169-4

**Published:** 2024-03-08

**Authors:** Peng Zhao, You Li, Xiangli Xu, Haobo Yang, Xintong Li, Shuai Fu, Zihong Guo, Jianing Zhang, Hairu Li, Jiawei Tian

**Affiliations:** 1 Department of Ultrasound, The Second Affiliated Hospital of Harbin Medical University, Harbin, 150001 China; 2 Department of Ultrasound, The Second Hospital of Harbin City, Harbin, 150001 China; 3 The Key Laboratory of Myocardial Ischemia, Ministry of Education, Harbin Medical University, Harbin, 150001 China; 4 Ultrasound Molecular Imaging Joint Laboratory of Heilongjiang Province, Harbin Medical University, Harbin, 150001 China

**Keywords:** Doxorubicin-induced myocardial toxicity, Ferroptosis, Neutrophil extracellular traps, YAP

## Abstract

Doxorubicin-induced cardiotoxicity (DIC), which is a cardiovascular complication, has become the foremost determinant of decreased quality of life and mortality among survivors of malignant tumors, in addition to recurrence and metastasis. The limited ability to accurately predict the occurrence and severity of doxorubicin-induced injury has greatly hindered the prevention of DIC, but reducing the dose to mitigate side effects may compromise the effective treatment of primary malignancies. This has posed a longstanding clinical challenge for oncologists and cardiologists. Ferroptosis in cardiomyocytes has been shown to be a pivotal mechanism underlying cardiac dysfunction in DIC. Ferroptosis is influenced by multiple factors. The innate immune response, as exemplified by neutrophil extracellular traps (NETs), may play a significant role in the regulation of ferroptosis. Therefore, the objective of this study was to investigate the involvement of NETs in doxorubicin-induced cardiomyocyte ferroptosis and elucidate their regulatory role. This study confirmed the presence of NETs in DIC in vivo. Furthermore, we demonstrated that depleting neutrophils effectively reduced the occurrence of doxorubicin-induced ferroptosis and myocardial injury in DIC. Additionally, our findings showed the pivotal role of high mobility group box 1 (HMGB1) as a critical molecule implicated in DIC and emphasized its involvement in the modulation of ferroptosis subsequent to NETs inhibition. Mechanistically, we obtained preliminary evidence suggesting that doxorubicin-induced NETs could modulate yes-associated protein (YAP) activity by releasing HMGB1, which subsequently bound to toll like receptor 4 (TLR4) on the cardiomyocyte membrane, thereby influencing cardiomyocyte ferroptosis in vitro. Our findings suggest that doxorubicin-induced NETs modulate cardiomyocyte ferroptosis via the HMGB1/TLR4/YAP axis, thereby contributing to myocardial injury. This study offers a novel approach for preventing and alleviating DIC by targeting alterations in the immune microenvironment.

**Supplementary Information:**

The online version contains supplementary material available at 10.1007/s00018-024-05169-4.

## Introduction

With the advancement of medical technology, there have been gradual improvements in the survival rates of patients with malignancies. Complications during treatment and long-term quality of life are a matter of concern [1]. Doxorubicin (DOX), which is a representative anthracycline, is extensively used to treat many malignancies, such as pediatric tumors, leukemia, and breast cancer [2]. DOX has significantly improved the long-term survival rates of patients with cancer, but these advances have been accompanied by significant cardiovascular complications. Due to the finite regenerative capacity of cardiomyocytes, they are particularly susceptible to the long-term effects of cytotoxic agents such as DOX [3]. One study revealed that 10% of patients developed fatal cardiomyopathy up to 15 years after treatment with DOX [4]. It has also been suggested that pediatric leukemia patients treated with DOX exhibit an eightfold increase in susceptibility to cardiovascular disease in adulthood compared to healthy children [5]. This not only imposes limitations on the use of DOX but also significantly impacts patient quality of life and even their survival following malignant tumor remission. Therefore, it is important to investigate the mechanisms underlying doxorubicin-induced cardiotoxicity (DIC) and identify targets for preventing or mitigating this condition.

Ferroptosis, which is a newly discovered form of programmed cell death, plays an important role in pathophysiological conditions during cardiac injury. DOX can directly impact iron homeostasis in cardiomyocytes through various mechanisms, resulting in oxidative stress and mitochondrial dysfunction [6, 7]. Furthermore, ferroptosis is the predominant form of regulated cell death in DIC [8]. Dexrazoxan, an iron chelating agent, is the sole FDA-approved clinical drug for treating DIC, thereby affirming the pivotal role of iron metabolism disorders in this context [9, 10]. Relevant experimental studies conducted by our team have also provided evidence for the involvement of ferroptosis in DIC, and changes in yes-associated protein (YAP) further suggested that the Hippo pathway played a certain role in DIC induced ferroptosis in cardiomyocytes [11]. However, the underlying mechanism through which DOX modulates the alterations in YAP expression remains unknown.

Alterations in the immune microenvironment play pivotal roles in the progression of myocardial injury [12]. DOX-induced tissue damage is closely associated with alterations in the immune microenvironment. Clayton Z. et al. revealed that DOX-induced aortic sclerosis was primarily driven by TNFα-mediated inflammation and extracellular matrix remodeling [13]. Bhagat A et al. demonstrated a significant increase in neutrophil levels in the cardiac tissue of mice treated with DOX, which mediated acute cardiac damage induced by DOX [14]. These findings suggest that neutrophils can modify the immune microenvironment and mediate myocardial damage. As a crucial component of the human innate immune system, neutrophils are the initial responders that are promptly activated and infiltrate tissue in response to stimulation [15]. Neutrophil extracellular traps (NETs) are a novel way for neutrophils to play a defensive role and were discovered in the past decade. The structure of NETs consists of a DNA reticular skeleton and intracellular proteins, however, this formation exhibits a dualistic nature [16, 17]. The release of cellular contents in NETs, such as neutrophil elastase (NE), myeloperoxidase (MPO), and high mobility group box-1 (HMGB1), can induce alterations in the immune microenvironment, thereby inducing more robust inflammatory responses and even leading directly to cellular damage [18]. Intriguingly, some scholars have found that elevated levels of circulating NETs markers following the initial administration of DOX chemotherapy were associated with an increased risk of subsequent cardiotoxicity [19]. These findings not only suggest that circulating indicators of NETs could serve as early biomarkers for predicting DIC but also indicate the involvement of NETs in regulating DIC development. Zhang H et al. reported that neutrophils and NETs played crucial roles in the pathogenesis of sepsis-induced acute lung injury by facilitating ferroptosis in alveolar epithelial cells [20]. Investigating the association between NETs and ferroptosis in DIC holds significant scientific merit and warrants further exploration.

This study confirmed the presence of NETs in DIC through animal experiments, and the release of HMGB1 subsequent to NETs formation could mediate cardiomyocyte ferroptosis via the Hippo pathway, thereby contributing to the development of DIC.

## Materials and methods

### Animal experiments

Male C57BL/6N mice (8–10 weeks old) were purchased from the Second Affiliated Hospital of Harbin Medical University. All animal care and use procedures were performed according to the Principles of Animal Care provided by the National Society for Medical Research and the Guide for the Care and Use of Laboratory Animals (Institute of Laboratory Animal Resources, NIH). The animal study protocol was conducted in accordance with the guidelines of the Research Ethics Committee of the Second Affiliated Hospital of Harbin Medical University, China (SYDW2022-071).

The mice were maintained in a specific pathogen-free environment at the animal facility of the Second Affiliated Hospital of Harbin Medical University for at least one week prior to the initiation of this experiment. The experimental animals were randomly divided into several groups. To investigate the inflammatory response and ferroptosis in DIC, we established a model by intraperitoneally injecting 6 mg/kg DOX (S1208, Selleck, USA) once per week for 4 weeks, resulting in a cumulative dose of 24 mg/kg (DOX). The control group was injected with the same volume of PBS at the same time points (CON). To evaluate the role of ferroptosis in DIC, the mice were pretreated with 20 mg/kg erastin (S7242, Selleck, USA) or 10 mg/kg ferrostatin-1 (Fer-1, HY-100579, MCE, USA) by intraperitoneal injections one day before DOX injection (DOX + erastin and DOX + Fer-1). To examine the impact of neutrophil depletion on DIC, 200 µg of the neutralizing antibody anti-Ly6G (Clone 1A8; BioXcell, USA) or 4 mg/kg GSK484 (HY-100514; MCE, USA), a type of protein arginine deiminase 4 inhibitor (PAD4−), was administered to the mice through the tail vein 24 h after each DOX injection (DOX + Anti-Ly6G and DOX + PAD4−). Similarly, the same dose of anti-Ly6G or GSK484 was administered after PBS injections (anti-Ly6G and PAD4−) for comparison. Additionally, one group received a combination of anti-Ly6G and Fer-1 following each DOX administration (DOX + Anti-Ly6G + Fer-1). To examine the function of HMGB1, mice treated with DOX and anti-Ly6G were intravenously administered 300 µg/kg recombinant mouse HMGB1 (P5532; Wuhan Fine Biotech, China) 24 h after each administration of anti-Ly6G (DOX + Anti-Ly6G + rHMGB1).

### Echocardiography

One week after the last injection, the mice were anesthetized with 1.25% tribromoethanol, the chest hair was removed by shaving, and echocardiography was performed with a Samsung RS80A Ultrasound System (Samsung Medison Co., Ltd., Seoul, Korea) equipped with a 7–12 MHz linear array transducer. Two-dimensional (2D) images of the left ventricle in the short-axis view were acquired with the parasternal approach. M-mode images of the left ventricle were acquired at the level of the papillary muscle following the guidance of the 2D image. The left ventricle ejection fraction (LVEF), left ventricular fraction shortening (LVFS) and left ventricular internal diameter in systole and diastole (LVIDs and LVIDd, respectively) were assessed by the M-mode from the average of three independent cardiac cycles. The operations were performed by an experienced physician who was blinded to the group allocations.

### Blood sample and heart tissue collection

After echocardiography, the mice were maintained under anesthesia by CO_2_ inhalation and underwent laparotomy and thoracotomy. Blood samples were collected from the inferior vena cava. The plasma was centrifuged, rapidly frozen in liquid N2 and subsequently stored at −80 °C for follow-up experiments.

The heart was excised following perfusion with PBS and sectioned along the short axis of the left ventricle. Half of the heart was fixed in 4% paraformaldehyde for histological analysis, and the other half was frozen in liquid N2 and cryopreserved at −80 °C for other experiments.

### Histopathology

For histological analysis, the harvested heart tissues were fixed in 4% paraformaldehyde for 72 h, dehydrated, cleared, paraffin-embedded, and sliced into 4-μm-thick sections for hematoxylin and eosin (H&E) staining and Sirius red staining. Morphological changes in cardiac tissues, such as inflammatory infiltration and fibrotic areas, were evaluated under an optical microscope (Olympus, Tokyo, Japan) to assess pathological conditions.

### Transmission electron microscopy

Small cubes of myocardial tissue (≤ 1 mm^3^) were rapidly excised from the left ventricle and washed with precooled PBS. Subsequently, the tissues were immediately fixed overnight at 4 °C in a 2.5% glutaraldehyde solution buffered with phosphate (pH 7.4). After being embedded, the samples were sliced into ultrathin Sects. (50 nm) and stained with uranyl acetate and lead citrate before being observed using an electron microscope (HITACHI, H-7650, Japan).

### Immunohistopathology

For immunohistochemical staining (IHC), heart tissue sections were subjected to antigen retrieval with citrate-EDTA antigen retrieval solution (P0086; Beyotime, China) in a 95–100 °C water bath for 15 min after dewaxing and rehydration. The subsequent steps included inhibiting endogenous peroxidase activity and permeabilization with 0.1% Triton X-100 solution for 15 min. After being blocked with 5% bovine serum albumin at room temperature for 30 min, the sections were incubated overnight at 4 °C with the following primary antibodies: Ly6G (1:500, ab238132, Abcam), HMGB1 (1:350, ab79823, Abcam), YAP (1:200, A1002, ABclonal), and phospho-YAP-S127 (1:100, AP0489, ABclonal). The sections were thoroughly washed with PBS, and subsequently incubated with an HRP-labeled goat anti-rabbit IgG secondary antibody (PV-6001; ZSGB-bio, Beijing, China) for 20 min at 37 °C. A DAB Peroxidase Substrate Kit (ZLI-9018; ZSGB-bio, Beijing, China) was used to visualize the positive region. Following hematoxylin staining of the nucleus, the sections were subjected to ethanol dehydration and xylene clearing. The sections were imaged with an optical microscope (Olympus, Tokyo, Japan) and then analyzed using ImageJ (NIH V1.8.0.112).

### Immunofluorescence analysis

To examine NETs formation in tissues, immunofluorescence (IF) analysis was performed. Heart tissues were embedded in optimal cutting temperature (OCT) compound (Tissue-Tek; Sakura Finetek, USA, CA) and cut to a thickness of 7 μm. The sections were fixed with 4% paraformaldehyde for 15–20 min, washed with PBS and blocked with 5% bovine serum albumin at room temperature for 30 min. Subsequently, the sections were incubated with primary antibodies against MPO (1:50, ab300650, Abcam) and Cit-H3 (1:100, ab219406, Abcam) overnight at 4 °C. Then, the sections were incubated with Alexa Fluor 555-conjugated anti-rabbit (1:400) and Alexa Fluor 488-conjugated anti-rat (1:400) secondary antibodies for 1 h at room temperature in the dark. Finally, to stain the nucleus and NETs skeleton, the sections were incubated with 10 µg/ml DAPI (A1013, Alphabio, Tianjin). The sections were observed with a fluorescence microscope (Leica, Wetzlar, Germany), and images were captured at 200X magnification.

### Cell culture and treatments

H9c2 cells were obtained from the BeNa Culture Collection (Beijing, China) and cultured in Dulbecco’s modified Eagle’s medium (HyClone, USA) supplemented with 10% fetal bovine serum (FBS) in a humidified atmosphere of 5% CO_2_ and 95% O_2_ at 37 °C. To validate that DOX induced ferroptosis, after the concentrations were screened by an activity assay, the cells were cultured with 1 μM Dox (S1208, Selleck, USA) for 24 h to establish the DIC model, and another group of cells was treated with 5 μM erastin (S7242, Selleck, USA) for 24 h to induce ferroptosis as a positive control. To examine the inhibition of ferroptosis, cells were pretreated with 10 μM Fer-1 (HY-100579, MCE, USA) for 1 h, followed by DOX treatment for another 24 h. The control group did not receive any treatment. To further investigate the regulatory role of HMGB1 and its receptor TLR4 in DIC, the cells were incubated with 1 μg rHMGB1 (P5532, Wuhan Fine Biotech, China) or 50 μM TAK242 (HY-11109, MCE, USA) or 1 μg rHMGB1 and 50 μM TAK242 in combination with 1 μM DOX for 24 h.

### Analysis of cell viability and LDH levels

After treatment with the different agents, cell viability was measured by using cell counting kit-8 (CCK-8) (CK04, Dojindo, Japan). H9c2 cells were seeded into 96-well plates, after which 10 μl of CCK-8 working solution was added to each well. Subsequently, the plates were incubated at 37 °C for 2 h. The absorbance was measured at 450 nm with a microplate reader (Tecan, Switzerland). The release of lactate dehydrogenase (LDH) was measured using a cytotoxicity LDH Assay Kit-WST (CK12, Dojindo, Japan) to assess the extent of cellular damage. The reagent was added in accordance with the manufacturer's instructions, and the absorbance was promptly measured at 490 nm.

### ELISA

The levels of the inflammatory cytokines TNF-α, IL-6, CXCL-1 and IL-8 in serum and tissues were measured by using ELISA kits (Jingkang Bioengineering Co., China) according to the manufacturer’s protocol. Serum levels markers of myocardial damage, including brain natriuretic peptide (BNP) and lactate dehydrogenase (LDH), were quantified using ELISA kits (Jingkang Bioengineering Co., China) according to the manufacturer’s instructions. NETs in serum and tissues were quantified by measuring the levels of the MPO-DNA complex and the NE-DNA complex using specialized ELISA kits (Meimian Industrial Co., China) according to the manufacturer’s protocol.

### Determination of MDA, ferrous ion and GSH levels

To evaluate the occurrence of ferroptosis in heart tissue, freshly isolated heart tissue was lysed to prepare tissue homogenates. Malondialdehyde (MDA) levels in heart tissues and H9c2 cells were measured by using a commercial kit (S0131; Beyotime, China) in accordance with the manufacturer’s instructions. Ferrous ion levels in heart tissues was quantified using an Iron Assay Kit (BC4355, Solarbio, Beijing) and those in H9c2 cells was quantified using an Iron Assay Kit (E1042, Alphabio, Tianjin) according to the manufacturer's instructions. The nonenzymatic antioxidant system in heart tissues and H9c2 cells was assessed by examining glutathione (GSH) and oxidized glutathione (GSSG) levels and the GSH/GSSG ratio using a GSH and GSSG Assay Kit (A061-1, Nanjing Jiancheng BioTech, China) according to the manufacturer's instructions.

### Western blotting

Protein was extracted from heart tissues and H9c2 cells with RIPA lysis buffer (P0013B; Beyotime Institute of Biotechnology, Shanghai, China) supplemented with a protease inhibitor and phosphatase inhibitor cocktail at 4 °C for 30 min, followed by centrifugation at 4 °C for 15 min. The BCA protein assay was used to determine the protein concentration. Protein samples (20 μg/group) were separated by 12% SDS‒PAGE and transferred onto PVDF membranes (Millipore; Burlington, MA, USA). After being blocked for 15–20 min at room temperature with blocking buffer (P0252, Beyotime Institute of Biotechnology, Shanghai, China), the membranes were incubated overnight at 4 °C with the following primary antibodies: GAPDH (1:2000, 5174S, CST), ACSL4 (1:2000, A6826, ABclonal), GPX4 (1:2000, A1933, ABclonal), HMGB1 (1:10,000, ab79823 Abcam), NE (1:2000, A13015, ABclonal), TLR4 (1:3000, A5258, ABclonal), YAP (1:2000, A1002, ABclonal), and phospho-YAP-S127 (1:500, AP0489, ABclonal). After being washed 3 times (5 min/wash) with TBST, the membranes were incubated with the appropriate horseradish peroxidase-conjugated secondary antibody (1:5000, 7074S, CST) at room temperature for 1 h. Finally, the protein bands were visualized with a supersensitive ECL chemiluminescence agent (MA0186, MeilunBio, Dalian). Band intensities were assessed with ImageJ software, and the protein expression levels were normalized to the GAPDH levels.

### Real-time quantitative PCR (RT–qPCR) analysis

Total RNA was isolated from heart tissue using an RNeasy Isolation Kit (DP419, Tiangen Biotech, China), and the RNA was reverse transcribed to cDNA with the miRcute Plus miRNA First-Strand cDNA Synthesis Kit (KR103, Tiangen Biotech, China). qPCR was performed with FS Universal SYBR Green Master Rox (Roche Diagnostics, Switzerland). The following primer pairs were used: GAPDH (forward, 5′-TGTGAACGGATTTGGCCGTA-3′; reverse, 5′-ACTGTGCCGTTGAATTTGCC-3′), Toll like receptor 2, TLR2 (forward, 5′-AGCCCATTGAGAGGAAAGCC-3′; reverse, 5′-CCAAAACACTTCCTGCTGGC-3′), Toll like receptor 4, TLR4 (forward, 5′-ACACCAGGAAGCTTGAATCCC-3′; reverse, 5′-AGGGACTTTGCTGAGTTTCTGA-3′) Receptor of Advanced Glycation Endproducts, RAGE (forward, 5′-CACGAGGATGAGGGCACCTA-3′; reverse, 5′-CCTCATCGCCGGTTTCTGTGA-3′). Gene expression levels were normalized to GAPDH levels to determine the relative expression levels, and the fold change was calculated using the 2^−ΔΔCT^ method.

### Statistical analysis

The data are presented as the mean ± standard deviation (SD). Group comparisons were performed by one-way ANOVA followed by the Bonferroni post hoc correction or unpaired *t* tests where appropriate. Statistical analysis and graphical representation were performed using GraphPad Prism v.9.0 Software (GraphPad, San Diego, CA, USA). *P* < 0.05 indicated statistical significance.

## Results

### Ferroptosis plays a pivotal role in the pathogenesis of cardiac damage associated with DIC

**Fig. 1:**
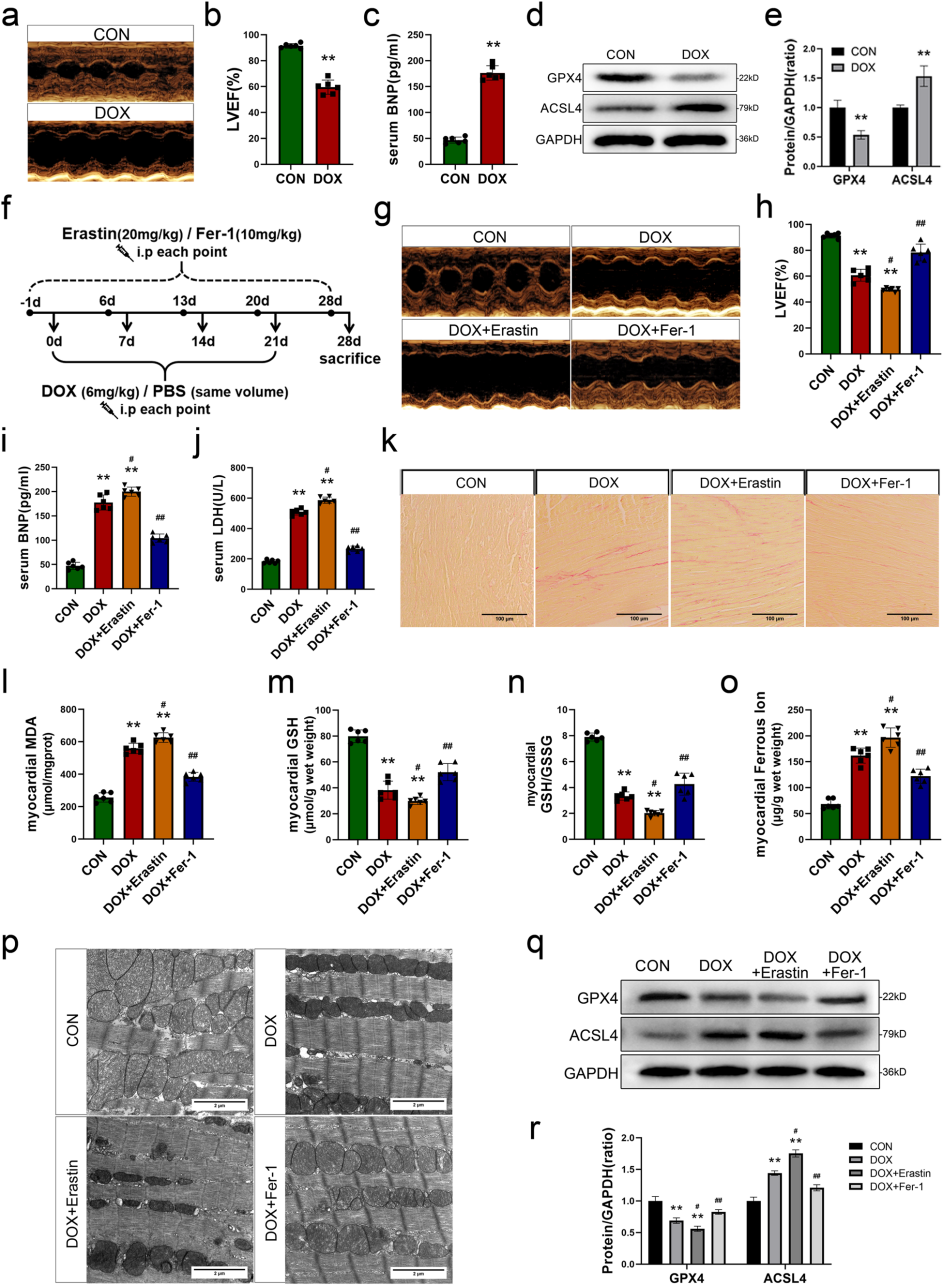
Ferroptosis plays a pivotal role in the pathogenesis of cardiac damage associated with DIC. **a** Representative images of M-mode echocardiography in DOX group and CON group. **b** Quantification of left ventricular ejection fraction (LVEF). **c** The levels of serum brain natriuretic peptide (BNP) after DOX injection. **d**, **e** Representative images and quantitative analysis of Western blotting analysis of GPX4 and ACSL4. **f** Experimental protocol of DOX, Erastin and Fer-1 jnjection. **g** Representative images of M-mode echocardiography in DOX group, DOX+Erastin group, DOX + Fer-1 group and CON group. **h** Quantification of left ventricular ejection fraction (LVEF) in each group. **i**, **j** The levels of serum brain natriuretic peptide (BNP) and lactate dehydrogenase (LDH). **k** Representative images of sirus red staining (scale bar, 200 μm). **i** Quantitative results of myocardial malondialdehyde (MDA) levels. **m**, **n** Quantitative results of myocardial  glutathione (GSH) levels and GSH/GSSG ratio. **o** The ferrous ion levels of myocardium in each group. **p** Representative images of mitochondria obtained by tissue transmission electron microscope (scale bar, 2 μm). **q**, **r** Representative images and quantitative analysis of Western blotting analysis of GPX4 and ACSL4. Values represent the mean ± SD. *P < 0.05, **P < 0.01 vs. CON group. ^#^*P* < 0.05, ^##^*P* < 0.01 vs. DOX group

We established a murine model of DIC by intraperitoneally administering DOX at regular intervals (Fig. S1a). After a 4-week treatment with DOX, we observed a significant reduction in the body weight of mice in the DOX group compared to the CON group (Fig. S1b), while a similar decrease was also noted in heart weight (Fig. S1c, d). The M-model echocardiography images revealed a dilatation of left ventricular which also reflected by the measurements of LVIDd and LVIDs (Figs. 1a and S1e, f). The LVEF and LVFS in the DOX group were also observed a significant decrease than those in the control group (Figs. 1b and S1g). Furthermore, the levels of serum BNP and LDH showed a significant increase in the DOX group compared with CON group (Figs. 1c and S1h). These results demonstrated the successful establishment of the DIC model. In addition, we quantified certain indicators of ferroptosis in the myocardium. The findings showed a significant increase in the concentration of MDA, a significant decrease in the levels of GSH and the ratio of GSH/GSSG, along with a notable increase in ferrous ion concentration within myocardium following DOX administration (Fig. S1i–l). Subsequently, western blot analysis was performed to determine the expression levels of the ferroptosis-related proteins GPX4 and ACSL4 in myocardial tissue, and the results revealed downregulation of GPX4 and upregulation of ACSL4 in the myocardial tissue of mice treated with DOX (Fig. 1d, e). These findings suggest that ferroptosis plays a role in myocardial injury following DOX administration.

To further determine the role of cardiomyocyte ferroptosis in DIC, we performed an in vivo experiment by administering the ferroptosis inducer erastin and the inhibitor Fer-1 to mice. The experimental design is shown in Fig. 1f. The results showed significant reductions in body weight and heart weight in DOX-treated mice, which was further exacerbated by erastin and ameliorated by Fer-1(Fig. S1m–o). The echocardiography results revealed that mice in the DOX + Erastin group exhibited more pronounced ventricular enlargement and a reduced amplitude of ventricular wall motion (Figs. 1g and S1p, q). Moreover, the LVEF and LVFS were lower in the DOX + Erastin treated group than in the DOX group. Conversely, the DOX + Fer-1 group exhibited significant alleviation in all echocardiographic indices compared to the DOX group (Figs. 1h and S1r). Serum levels of BNP and LDH in each group were quantified using ELISA kits. Results showed the DOX + Erastin group exhibited even higher levels of both BNP and LDH than the DOX group, whereas the DOX + Fer-1 group exhibited significantly lower levels than the DOX group (Fig. 1i, j). Sirius red staining revealed the presence of myocardial fibrosis in the DOX group, which was further exacerbated in the DOX + Erastin group. However, these detrimental effects were mitigated in the DOX + Fer-1 group compared to the DOX group (Figs. 1k and S1s). These findings suggest that Fer-1 effectively mitigates DOX-induced myocardial damage. Subsequently, the alteration of MDA, GSH levels and GSH/GSSG ratio, as well as ferrous ion content were also detected. Results revealed that compared to the DOX group, the DOX + Erastin group exhibited a more pronounced increase in MDA contents, accompanied by a decrease in GSH levels and GSH/GSSG ratio, as well as an significantly elevation in ferrous ion levels. However, the application of Fer-1 effectively reversed the alterations in these indicators (Fig. 1l–o). Mitochondrial morphology in myocardial tissue was examined using transmission electron microscopy. The results showed that the DOX group exhibited evident mitochondrial damage, which was characterized by reductions in size and increased membrane density. Compared to the DOX group, the DOX + Erastin group exhibited exacerbated mitochondrial damage, and treatment with Fer-1 significantly attenuated DOX-induced mitochondrial damage (Figs. 1p and S1t). Western blot analysis of the ferroptosis-related proteins GPX4 and ACSL4 showed that both DOX and erastin increased ferroptosis, whereas the administration of Fer-1 before DOX treated effectively alleviated ferroptosis in the myocardium (Fig. 1q, r). These results suggest that ferroptosis in the myocardium is a vital mechanism of DIC in mice.

### DIC mice demonstrates myocardium inflammation accompanied by the activation of NETs and the release of HMGB1

**Fig. 2:**
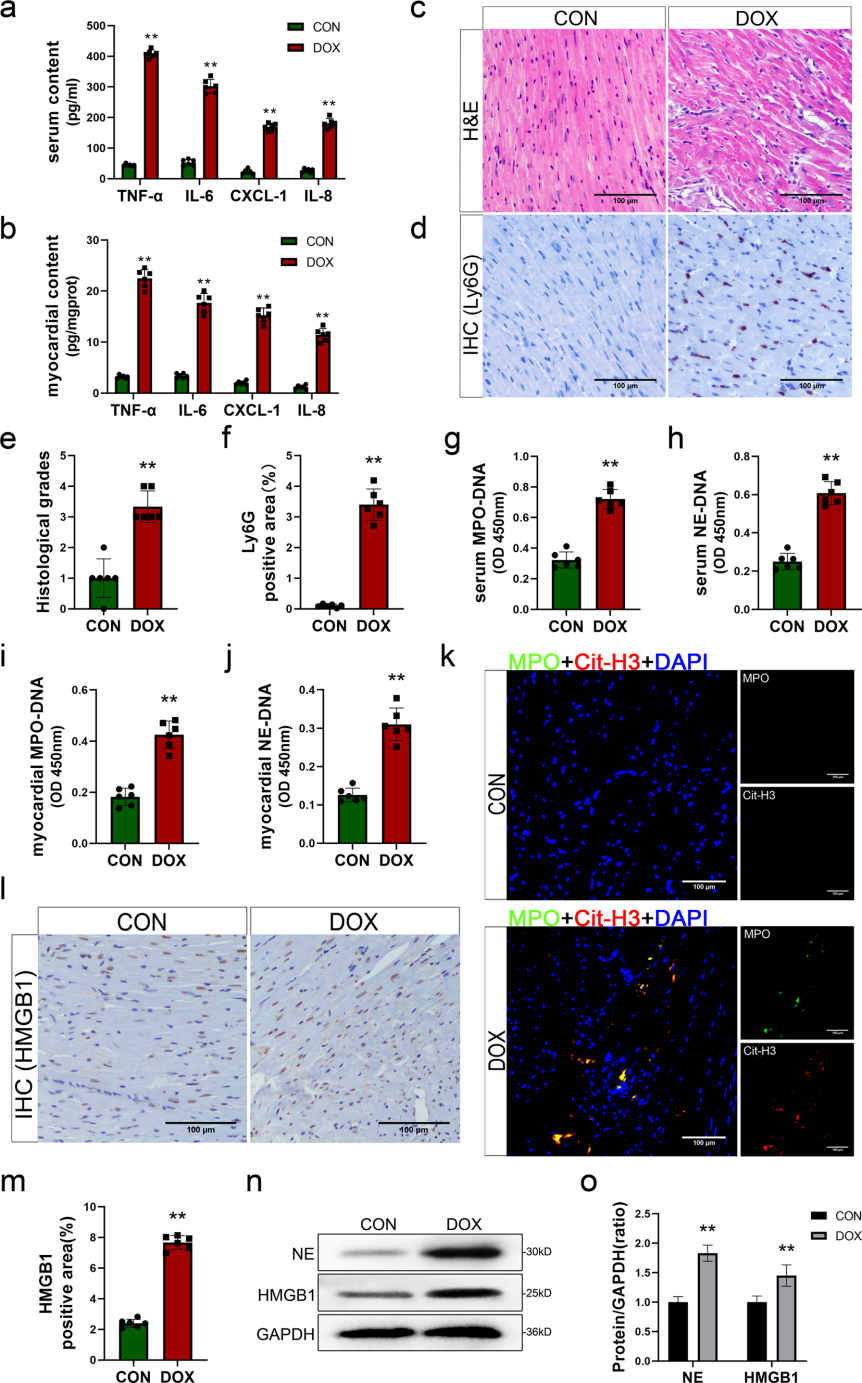
DIC mice demonstrates myocardium inflammation accompanied by the activation of NETs and the release of HMGB1. **a**, **b** The levels of cytokines TNF-α, IL-6, CXCL-1 and IL-8 in serum and myocardial content. **c** Representative images of H&E staining (scale bar, 200 μm). **d** Representative images and quantitative results of Ly6G immunohistochemical staining (scale bar, 200 μm). **e** Quantitative results of H&E staining analyzed based on histological grades. **f** Quantitative results of Ly6G immunohistochemical staining. **g**, **h**, **i**, **j** Quantitative analysis of MPO-DNA and NE-DNA complexes of serum and myocardial tissues. **k** Representative images of immunofluorescence staining of MPO (green), Cit-H3 (red) and DAPI (blue) to represented NETs formation (scale bar, 200 μm). **l**, **m** Representative images and quantitative results of HMGB1 immunohistochemical staining. **n**, **o** Representative images and quantitative analysis of Western blotting analysis of NE and HMGB1.Values represent the mean ± SD. *P < 0.05, **P < 0.01 vs*.* CON groupof MPO (green), Cit-H3 (red)

To examine the inflammatory response in mice after DOX stimulation, the levels of the cytokines TNF-α, IL-6, CXCL-1 and IL-8 in serum and heart tissue of mice in the DOX and control groups were examined by ELISA kits. The levels of inflammatory cytokines in serum and heart tissues were significantly increased in mice treated with DOX compared to those in the control group. (Fig. 2a, b). Moreover, myocardial tissue in the two groups was histologically examined by HE staining. In the DOX group, there was disorganized arrangement of myocardial cells, vacuoles, and increased infiltration of inflammatory cells in the interstitial space (Fig. 2c, e). These findings provide further evidence of myocardial inflammation in DIC. The primary function of the chemokines CXCL-1 and IL-8 is to facilitate neutrophil infiltration. Therefore, IHC staining for Ly6G was performed on myocardial tissue to assess the extent of neutrophil infiltration. The results showed a significant increase in  neutrophil infiltration in the myocardial tissue of mice in the DOX group (Fig. 2d, f). These findings confirmed that DOX induced additional neutrophil infiltration in myocardial tissue. Furthermore, we also investigated the neutrophil infiltration at various time points following administration of DOX and conducted combined depletion of neutrophils. Results revealed the levels of neutrophil-related chemokines IL-8 and CXCL-1 exhibited a gradual increase in direct proportion to the cumulative dose of DOX (Fig. S2a–d). This alteration was further substantiated by employing Ly6G IHC staining (Fig. S2e, f).

MPO-DNA and NE-DNA are reliable indicators of NETs and can be detected in both systemic circulation and target organs [21]. Therefore, to examine the occurrence of NETs in neutrophils induced by DOX stimulation, we used ELISA kits to measure the levels of MPO-DNA and NE-DNA in the circulation and myocardium in the two groups of mice. The results showed significant increases in the levels of MPO-DNA and NE-DNA in the circulation and myocardium of DOX-treated mice (Fig. 2g–j). To visualize NETs in myocardial tissue, immunofluorescence staining was performed by fluorescently labeling MPO and Cit-H3, and the determination of NETs occurrence was based on the colocalization of these factors with DNA. Immunofluorescence staining revealed that MPO and Cit-H3 were more abundant and colocalized with dense nuclei in the myocardium after DOX treatment (Fig. 2k), which confirmed the presence of NETs in myocardial tissue. Moreover, the occurrence of NETs was accompanied by the release of NE and HMGB1. We measured the expression of NE and HMGB1 in the myocardium by Western blotting and observed significant upregulation of these factors (Fig. 2n, o). Subsequently, IHC staining revealed that the expression of HMGB1 in the myocardium of the DOX group was significantly increased, and this effect was accompanied by positive staining outside the nucleus (Fig. 2l, m). This finding not only suggests that DOX induces neutrophil infiltration and the formation of NETs in myocardial tissue but also shows the diffusion of excessive HMGB1 through the interstitial space.

### Neutrophil depletion of DIC mice attenuates the production of NETs and the release of HMGB1

**Fig. 3:**
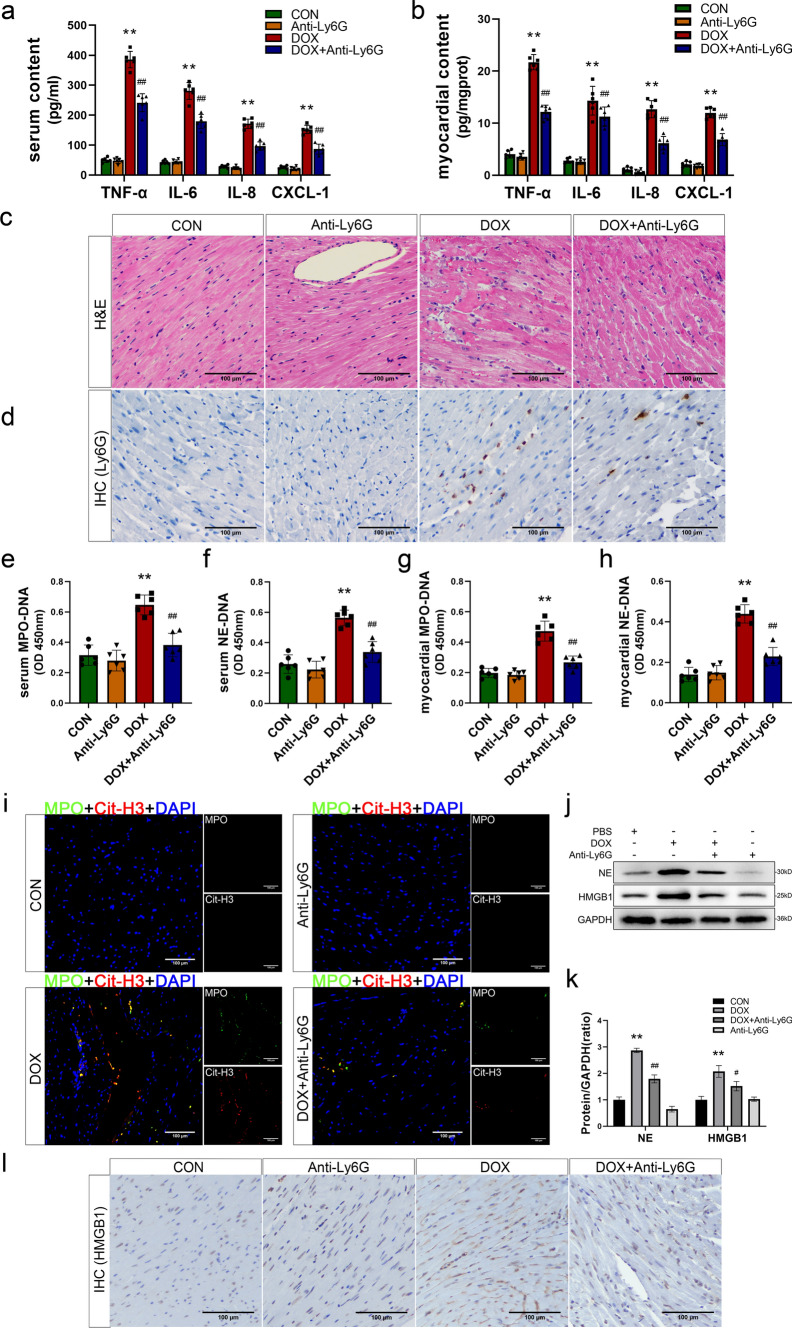
Neutrophil depletion of DIC mice attenuates the production of NETs and the release of HMGB1. **a**, **b** The content of cytokines TNF-α, IL-6, CXCL-1 and IL-8 in serum and myocardial tissues. **c**, **d** Representative images of H&E staining and Ly6G immunohistochemical staining (scale bar, 200 μm). **e**, **f**, **g**, **h** Quantitative analysis of serum and myocardial tissues MPO-DNA and NE-DNA complexes by ELISA kits. **i** Representative images of immunofluorescence staining of MPO (green), Cit-H3 (red) and DAPI (blue) to represented NETs formation (scale bar, 200 μm). **j**, **k** Representative Western Blot images and relative expression of NE and HMGB1. **l** Representative images of HMGB1 immunohistochemical staining (scale bar, 200 μm).Values represent the mean ± SD. *P < 0.05, **P < 0.01 vs. CON group. ^#^*P* < 0.05, ^##^*P* < 0.01 vs. DOX group.

To further investigate the involvement of NETs in ferroptosis during DIC, we employed anti-Ly6G neutralizing antibodies to deplete neutrophils, thereby effectively preventing the formation of NETs in mice following DOX stimulation, as shown in Fig. S3a. The therapeutic effect of anti-Ly6G antibody treatment on mitigating neutrophil infiltration was subsequently examined. The levels of cytokines, particularly CXCL-1 and IL-8, in the serum and myocardial tissue were significantly reduced in the mice treated with DOX and anti-Ly6G, which correlated with a decrease in neutrophil counts (Fig. 3a, b). Specifically, the chemotactic cytokines for neutrophils were significantly reduced, while TNF-α and IL-6 were unchanged. These findings suggested that the administration of anti-Ly6G antibodies reduced in neutrophil counts in mice treated with DOX. HE staining of myocardial sections was also revealed that the DOX + Anti-Ly6G group exhibited reduced vacuolization and edema in the myocardium, along with a significant decrease in inflammatory cell infiltration, compared to the DOX group (Figs. 3c and S3b). Compared with the control group, the anti-Ly6G group exhibited regular arrangement of the myocardium, and no significant changes were observed. These findings indicate that anti-Ly6G antibodies alone did not induce any pathological damage to cardiac tissue. The infiltration of neutrophils was further assessed by IHC staining, which revealed a significant reduction in Ly6G + neutrophil infiltration in the myocardial tissue of mice in the DOX + Anti-Ly6G group compared to those in the DOX group (Figs. 3d and S3c). This observation provides direct evidence for the effective depletion of DOX-induced neutrophils by the neutralizing anti-Ly6G antibody.

Furthermore, to explore the impact of neutrophil depletion on NETs development, we performed a comprehensive analysis of MPO-DNA and NE-DNA levels in the blood and myocardial tissue of mice in each group. Treatment with the anti-Ly6G antibody effectively attenuated the increases in MPO-DNA and NE-DNA in the serum and myocardial tissue (Fig. 3e–h), suggesting that neutrophil depletion by these antibodies successfully inhibited NETs. Moreover, these changes were observed by immunofluorescence staining. The results revealed that the upregulation of MPO and Cit-H3 was accompanied by colocalization with nuclei in the myocardium of DOX-treated mice. However, this effect was significantly attenuated in the DOX + Anti-Ly6G group (Fig. 3i). The reduction in cell contents released with NETs was further examined by Western blot analysis, which showed significantly lower levels of NE and HMGB1 in the DOX + Anti-Ly6G group than in the DOX group (Fig. 3j, k). Moreover, IHC staining of HMGB1 in the myocardium showed a significant decrease in HMGB1 expression in the DOX + Anti-Ly6G group, which was particularly evident in the positive area within the myocardial gap (Figs. 3l and S3d). Collectively, these findings provide suggest that anti-Ly6G antibody-mediated neutrophil depletion effectively reduces neutrophil levels in myocardial tissue following DOX stimulation, thereby inhibiting NETs formation and attenuating HMGB1 release into the interstitial space.

### Inhibition of NETs alleviates doxorubicin-induced myocardial injury by reducing ferroptosis

**Fig. 4:**
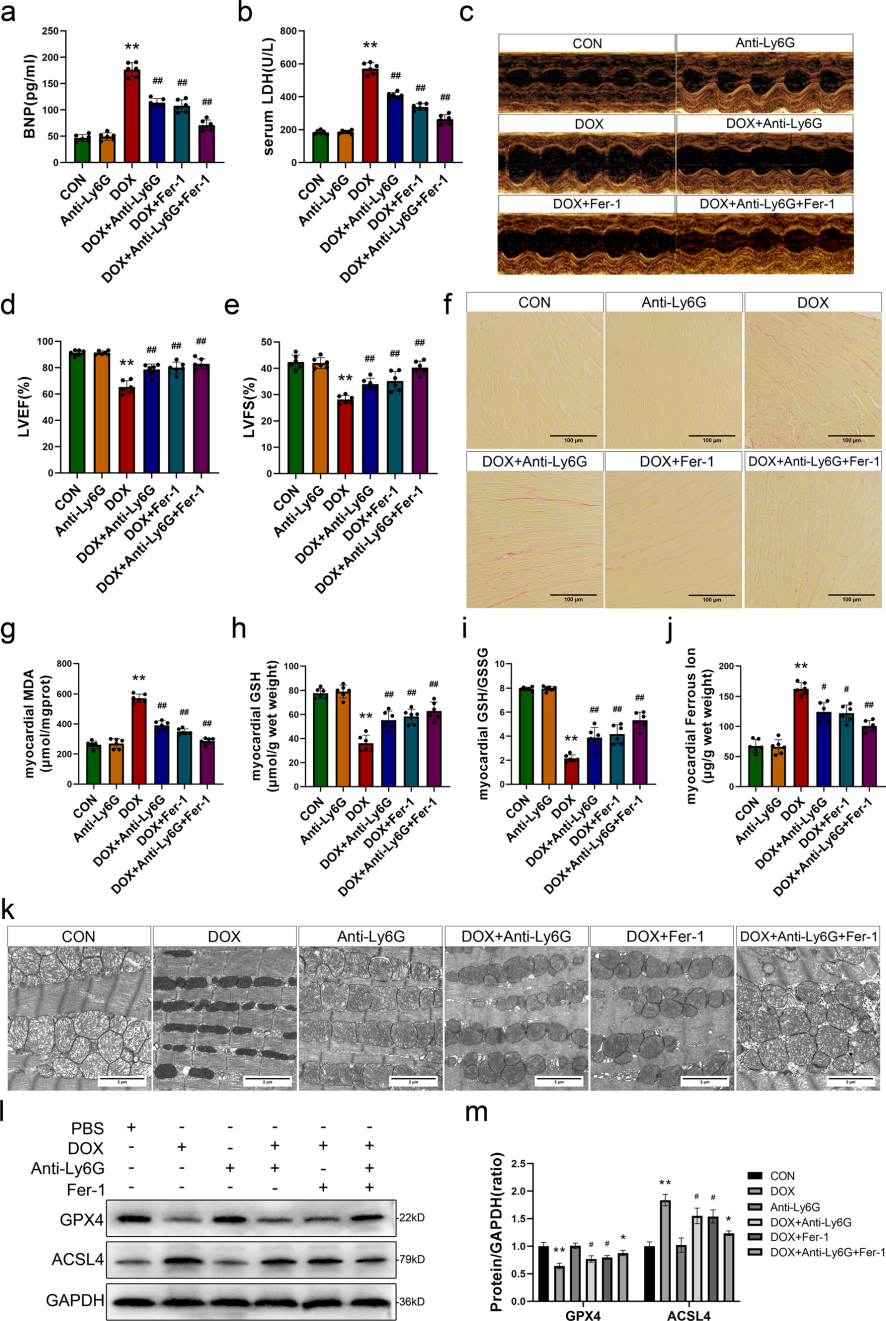
Inhibition of NETs alleviates doxorubicin-induced myocardial injury by reducing ferroptosis. **a**, **b** The brain natriuretic peptide (BNP) and lactate dehydrogenase (LDH) levels in serum. **c** Representative images of M-mode echocardiography. **d**, **e** Quantification of left ventricular ejection fraction (LVEF) and left ventricular fraction shortening(LVFS). **f** Representative images of sirus red staining (scale bar, 200 μm). **g** The levels of malondialdehyde (MDA) in myocardium. **h**, **i** Quantitative results of myocardial glutathione (GSH) levels and GSH/GSSG ratio. **j** The quantification of ferrous iron in myocardium. **k** Representative images of mitochondria obtained by tissue transmission electron microscope (scale bar, 2 μm). **l**, **m** Representative images and quantitative analysis of Western blotting analysis of GPX4 and ACSL4.Values represent the mean ± SD. *P < 0.05, **P < 0.01 vs. CON group. ^#^*P* < 0.05, ^##^*P* < 0.01 vs. DOX group

To investigate the effects of NETs inhibition on reducing DOX-induced ferroptosis and myocardial damage, we combined the ferroptosis inhibitor Fer-1 with anti-Ly6G to assess alterations in cardiac function and indicators associated with ferroptosis in experimental animals. The results showed that body weight and heart weight loss were improved in mice treated with DOX after neutrophil depletion, and the changes were similar to those observed in the Fer-1 treatment group (Fig. S4a, b). Additionally, compared with those in the DOX group, serum BNP and LDH levels in the DOX + Anti-Ly6G and DOX + Fer-1 groups were reduced (Fig. 4a, b), indicating that NETs inhibition and Fer-1 treatment could alleviate myocardial injury and heart failure induced by DOX. Moreover, M-mode echocardiography revealed that, compared to those in the DOX group, mice in the DOX + Anti-Ly6G and DOX + Fer-1 groups exhibited a reduced ventricular diameter and improved myocardial motion (Figs. 4c and S4c, d). Additionally, the LVEF and LVFS values were significantly higher than those observed in mice stimulated with DOX. The level in the Anti-Ly6G group was comparable to that in the CON group (Fig. 4d, e). Sirius red staining revealed the absence of myocardial fibrosis in the CON group and the Anti-Ly6G group. Conversely, mice in the DOX group exhibited significantly increased collagen fiber levels in myocardial tissue. However, mice treated with anti-Ly6G or Fer-1 showed substantial reductions in myocardial fibrosis compared to those in the DOX group. These improvements were more pronounced when anti-Ly6G was combined with Fer-1 (Figs. 4f and S4e). These findings suggest that, similar to ferroptosis inhibitors, the inhibition of NETs can effectively ameliorate DOX-induced cardiac functional impairment, and treatment with anti-Ly6G antibodies alone does not impact cardiac function in mice.

Although we confirmed that NETs inhibition may alleviate cardiac dysfunction in DIC, further evidence was needed to determine whether DOX-induced cardiac damage was ameliorated by reducing ferroptosis in cardiomyocytes. Therefore, we assessed the concentrations of aforementioned ferroptosis indicators in myocardial tissue. Similar to those in the DOX + Fer-1 group, MDA levels in the myocardium in the DOX + Anti-Ly6G group were significantly lower than those in the DOX group (Fig. 4g). Additionally, increases in the GSH concentration and GSH/GSSG ratio were also observed in both DOX + Anti-Ly6G and DOX + Fer-1 group (Fig. 4h, i). These findings indicate that inhibiting NETs can effectively reduce the increase in lipid peroxidation induced by DOX. In addition, the DOX + Anti-Ly6G group and DOX + Fer-1 group exhibited decreased levels of ferrous ions in the myocardium compared to the DOX group (Fig. 4j). No significant difference was observed between the Anti-Ly6G group and the CON group of these indicators. Myocardial mitochondria in the CON group and Anti-Ly6G group were observed by transmission electron microscopy. In the CON and Anti-Ly6G groups, the volume of myocardial mitochondria appeared normal, and there were intact membranes and clear internal ridge structures. Notably, mitochondria in the DOX group showed significant shrinkage and reduced numbers, which were accompanied by an overall increase in density and an indistinct internal structure. However, the characteristic ultrastructural damage associated with ferroptosis was significantly reduced in the DOX + Anti-Ly6G and DOX + Fer-1 groups (Figs. 4k and S4f). Western blot analysis of GPX4 and ACSL4 in the myocardium in each group revealed that, compared to that in the DOX group, GPX4 protein expression was upregulated, while ACSL4 protein expression was downregulated in the DOX + anti-Ly6G group (Fig. 4l, m), which was consistent with the changes observed in the DOX + Fer-1 group. Similarly, these improvements in characteristic ferroptosis markers were more pronounced in mice treated with anti-Ly6G + Fer-1. This strongly suggests that inhibiting NETs alleviates ferroptosis in cardiomyocytes induced by DOX.

To further validate the effect of NETs inhibition on mitigating DIC-induced ferroptosis and cardiac dysfunction, we administered another NETs inhibitor in vivo. PAD4 is a pivotal enzyme in the formation of NETs. GSK484, which is a specific PAD4 inhibitor, was used to assess alterations in cardiac function and ferroptosis markers in mice. We initially evaluated aforementioned inflammatory cytokines and NETs markers both in serum and myocardial tissue to determine the inhibitory effects of GSK484. The findings demonstrated that the inhibition of PAD4 effectively attenuated the levels of NETs markers, while exhibiting no significant impact on serum inflammatory cytokines TNF-α, IL-8, IL-6, CXCL-1 (Fig. S5a-f). Furthermore, we found that the PAD4 inhibitor could enhance cardiac function injury caused by DOX, as evidenced by echocardiography, heart weight, as well as serum BNP and LDH analysis (Fig. S5g–k). Furthermore, analysis of MDA and GSH levels, the GSH/GSSG ratio, ferrous ion levels (Fig. S5l–o), and myocardial expression of ferroptosis-related proteins (Fig. S5p) indicated that the PAD4 inhibitor acted as a potent NETs inhibitor to attenuate DOX-induced ferroptosis.

### HMGB1 serve as a crucial regulatory factor in the reduction of ferroptosis mediated by NETs inhibition in DIC

**Fig. 5:**
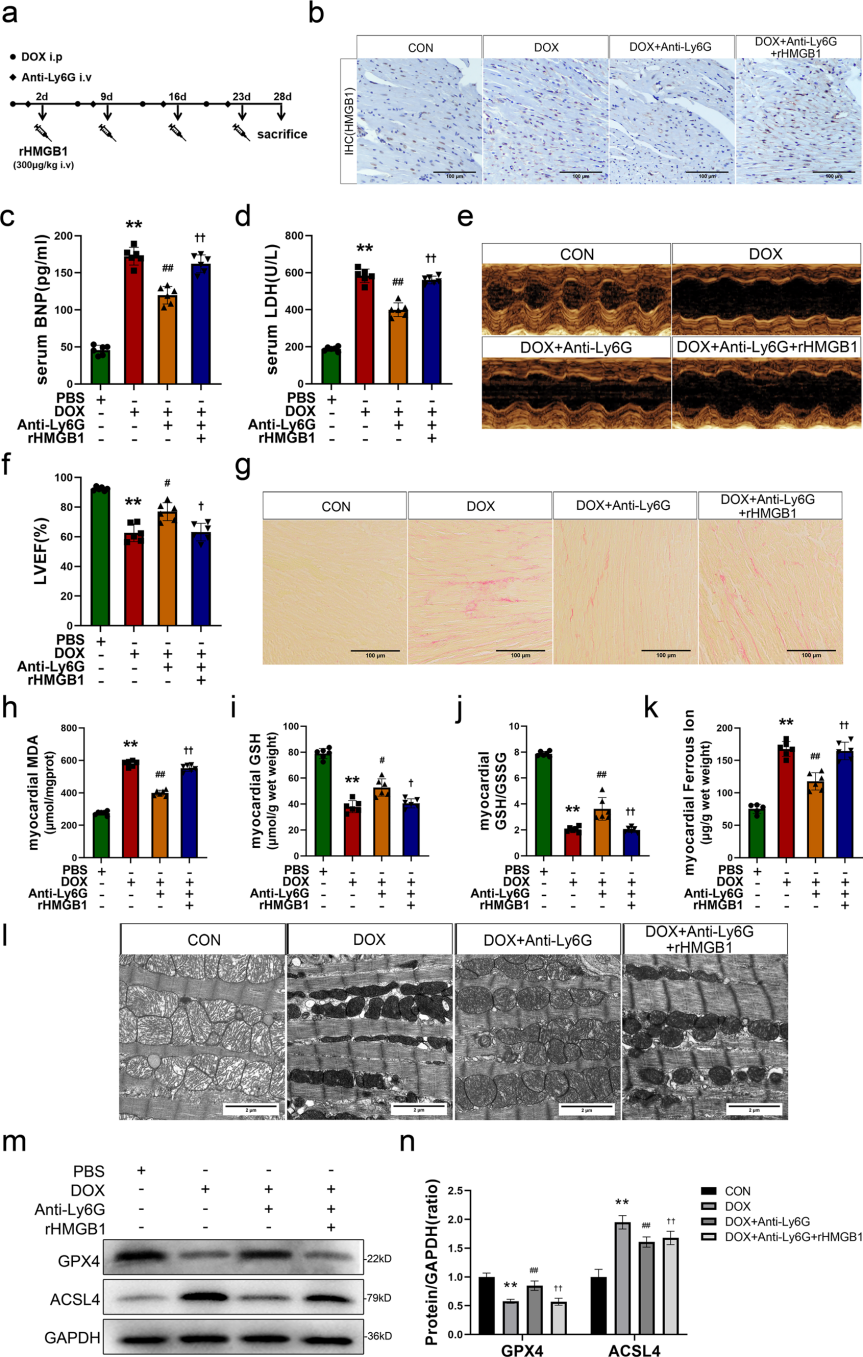
HMGB1 serve as a crucial regulatory factor in the reduction of ferroptosis mediated by NETs inhibition in DIC. **a** Experimental protocol. **b** Representative images of HMGB1 immunohistochemical staining after rHMGB1 injected (scale bar, 200 μm). **c**, **d** The brain natriuretic peptide (BNP) and lactate dehydrogenase (LDH) levels in serum. **e**, **f** Representative images of M-mode echocardiography and Quantification of left ventricular ejection fraction (LVEF) after rHMGB1 injected. **g** Representative images of sirus red staining (scale bar, 200 μm). **h** Quantitative results of malondialdehyde (MDA) levels in myocardial tissues. **i**, **j** Quantitative results of myocardial glutathione (GSH) levels and GSH/GSSG ratio. **k** The Quantification of ferrous iron levels in myocardium. **l** Representative images of mitochondria obtained by tissue transmission electron microscope (scale bar, 2 μm). **m**, **n** Representative images and quantitative analysis of Western blotting analysis of GPX4 and ACSL4.Values represent the mean ± SD. *P < 0.05, **P < 0.01 vs. CON group. ^#^*P* < 0.05, ^##^*P* < 0.01 vs. DOX group. ^†^*P* < 0.05, ^††^*P* < 0.01 vs. DOX + Anti-Ly6G group

The aforesaid findings showed that inhibiting NETs can effectively ameliorate myocardial injury and ferroptosis in DIC. The in vivo process underlying this phenomenon is intricate, necessitating further exploration of the molecular players involved. Given its role as a crucial DAMP, extracellular HMGB1 actively participates in the inflammatory response and tissue injury. Therefore, recombinant HMGB1 was administered to neutrophil-depleted mice following DOX stimulation (as shown in Fig. 5a), and cardiac function injury and ferroptosis were examined to determine whether HMGB1 is a key molecule in the NETs-mediated regulation of DIC.

IHC staining of HMGB1 in the myocardium of mice from each group revealed that the reduction in HMGB1 levels due to anti-Ly6G treatment was reversed by the administration of rHMGB (Figs. 5b and S6a). We found that the decrease in the serum BNP and LDH levels induced by neutrophil depletion was reversed in the rHMGB1 group (Fig. 5c, d). Similarly, the weight of heart tissue in mice subjected to rHMGB1 treatment further decreased (Fig. S6b, c). Neutrophil depletion mitigated the increase in left ventricular diameter and attenuated the impairment of wall motion in mice following DOX stimulation. However, treatment with rHMGB1 further exacerbated wall motion impairment and increased the LVIDd and LVIDs, as evaluated by M-mode echocardiography (Figs. 5e and S6d, e). Furthermore, statistical analysis of left ventricular systolic function showed reductions in LVEF and LVFS in the DOX + Anti-Ly6G + rHMGB1 group compared to the DOX + Anti-Ly6G group (Figs. 5f and S6f). Sirius red staining revealed a significant reduction in collagen fiber levels in the myocardial tissue of mice in the DOX + Anti-Ly6G group compared to the DOX group, and exacerbated myocardial fibrosis was observed in mice in the DOX + Anti-Ly6G + rHMGB1 group (Figs. 5g and S6g). These findings suggest that HMGB1 plays a crucial role in the regulation of NETs during DOX-induced cardiac dysfunction. Next, a series of experiments were performed to determine the impact of HMGB1 on alterations in cardiac function by affecting ferroptosis. The administration of rHMGB1 increased MDA levels and decreased GSH levels and the GSH/GSSG ratio in the myocardial tissues of DOX- and Anti-Ly6G-treated mice (Fig. 5h–j). These results indicate that the amelioration of lipid peroxidation induced by DOX due to neutrophil depletion was abrogated in the myocardial tissue of mice treated with rHMGB1. Similarly, compared with those in the DOX + anti-Ly6G group, ferrous ion levels in the myocardial tissue of the mice in the DOX + Anti-Ly6G + rHMGB1 group were also increased, nearly reaching the same levels as those in the DOX group (Fig. 5k). Transmission electron microscopy was used to observe myocardial mitochondrial damage, and the extent of mitochondrial damage was less severe in the DOX + Anti-Ly6G group than in the DOX group. However, in the DOX + Anti-Ly6G + rHMGB1 group, mitochondrial ultrastructural damage characteristic of ferroptosis was exacerbated (Figs. 5l and S6h). Finally, the upregulation of the GPX4 protein and downregulation of the ACSL4 protein in the myocardial tissue of mice in the DOX + Anti-Ly6G + rHMGB1 group provided direct evidence that rHMGB1 injection exacerbated ferroptosis in cardiomyocytes treated with DOX and anti-Ly6G (Fig. 5m, n). These findings suggest that HMGB1 is a key regulatory molecule that mediates DOX-induced ferroptosis through NETs.

### HMGB1 released by NETs regulats YAP expression through TLR4

**Fig. 6:**
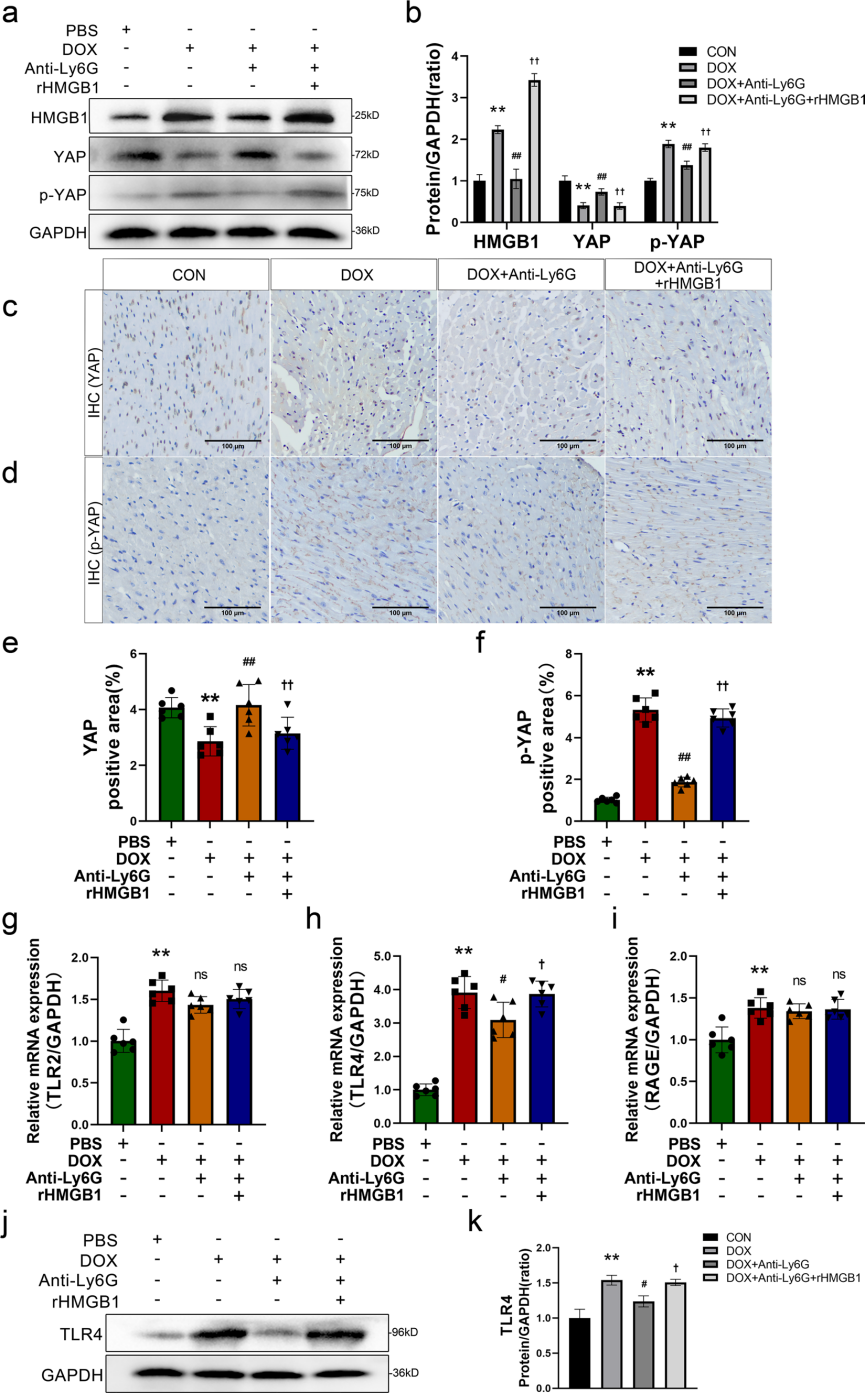
HMGB1 released by NETs regulats YAP expression through TLR4. **a**, **b** Western Blot was performed and quantitative analysed the relative expression of HMGB1, YAP and p-YAP. **c**, **d**, **e**, **f** Representative images and quantitative results of YAP and p-YAP immunohistochemical staining (scale bar, 200 μm). **g**, **h**, **i** Evaluate the expression of receptors TLR2,TLR4 and RAGE by RT-qPCR. **j**, **k** Representative images and quantitative analysis of Western blotting analysis of TLR4. Values represent the mean ± SD. *P < 0.05, **P < 0.01 vs. CON group. ns indicates no significance, ^#^*P* < 0.05, ^##^*P* < 0.01 vs. DOX group. ns indicates no significance, ^†^*P* < 0.05, ^††^*P* < 0.01 vs. the DOX + Anti-Ly6G group

Our previous studies demonstrated that YAP plays a role in DOX-induced ferroptosis in myocardial tissue because YAP is susceptible to alterations in the extracellular environment. Therefore, we postulated that the alterations in the immune microenvironment of cardiomyocytes induced by the increase in extracellular HMGB1 affected the function of YAP. The protein expression of HMGB1, YAP, and phospho-YAP in mouse myocardial tissue was assessed by Western blot analysis. DOX upregulated HMGB1 expression, downregulated YAP expression, and upregulated phospho-YAP expression in the myocardial tissue of mice. These changes in protein expression were reversed by neutrophil depletion (Fig. 6a, b). However, subsequent injection of rHMGB1 abrogated these alterations. IHC staining of YAP and phospho-YAP in the myocardium in each group demonstrated a decrease in YAP expression, and an increase in phospho-YAP expression was observed in the DOX group and the DOX + anti-Ly6G + rHMGB1 group (Fig. 6c–f). These findings suggest a close association between alterations in HMGB1 and the protein expression of YAP, and an increase in HMGB1 levels can increase the phosphorylation of the YAP protein, thereby inducing the Hippo pathway. Therefore, we performed a preliminary investigation to determine the mechanism through which HMGB1 induces these alterations. HMGB1 typically binds to receptors such as TLR2/TLR4/RAGE on the myocardial cell membrane, initiating a cascade of signaling pathway alterations and thereby exerting biological regulatory effects. We assessed the mRNA levels of TLR2, TLR4, and RAGE in mouse myocardial tissue using RT‒qPCR. The results revealed a significant increase in TLR4 mRNA level in response to DOX stimulation, and neutrophil depletion effectively reversed this increase. Additionally, treatment with rHMGB1 re-elevated TLR4 mRNA levels (Fig. 6g–i). The protein expression of TLR4 was validated by Western blot analysis, which revealed significant upregulation in the DOX group and the DOX + anti-Ly6G + rHMGB1 group (Fig. 6j, k). These findings indicate that an increase in extracellular HMGB1 can impact cardiomyocyte ferroptosis by activating and binding to TLR4 on cardiomyocytes, thereby modulating the YAP protein and inducing the Hippo pathway.

### Inhibition of TLR4 alleviates ferroptosis induced by DOX and HMGB1 by regulating YAP in vitro

**Fig. 7:**
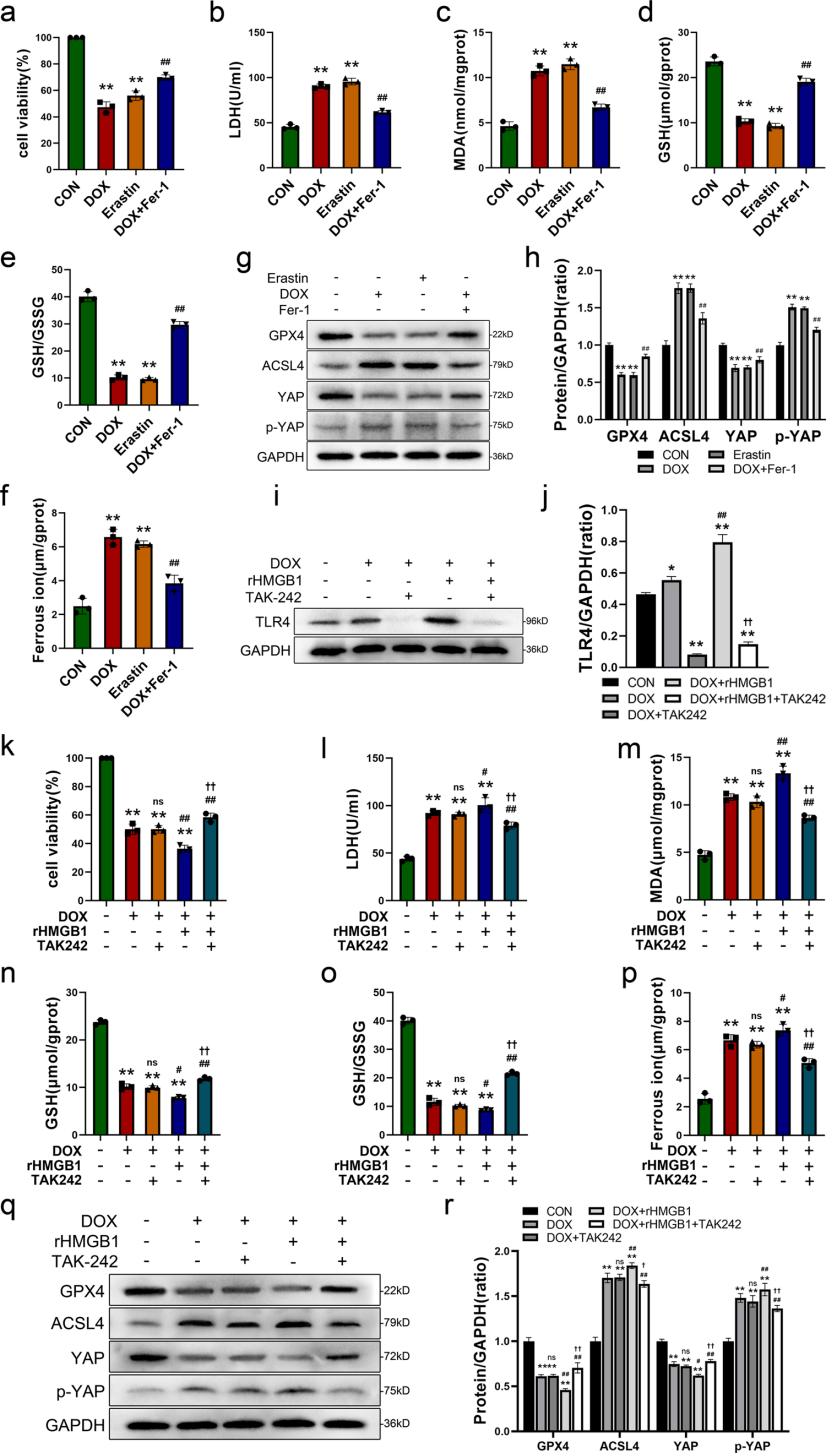
Inhibition of TLR4 alleviates ferroptosis induced by DOX and HMGB1 by regulating YAP in vitro. **a**, **b** The cell viability and lactate dehydrogenase (LDH) activity of H9c2 cells (treated with 1 µm DOX or 5 µm Erastin or pretreated with 10 μM Fer-1 for 1 h followed by DOX treatment for another 24 h. Fig. **c**–**h** were also obtained using this method). **c** Quantitative results of intracellular malondialdehyde (MDA) contents of H9c2 cells. **d**, **e** Quantitative results of intracellular glutathione (GSH) levels and GSH/GSSG ratio. **f** The quantification of intracellular ferrous iron. **g**, **h** Representative images and quantitative analysis of Western blotting analysis of GPX4, ACSL4, YAP and p-YAP in H9c2 cells after DOX, Erastin and Fer-1 treatment. **i**, **j** Representative images and quantitative analysis of Western blotting analysis of TLR4 in H9c2 cells (24 h after 1 µm DOX treatment and 1 μg rHMGB1 or 50 μM TAK242 or both of them co-incubated with 1 µm DOX for 24 h. Fig. **k**–**r** were also obtained using this method). **k**, **l** Cell viability and lactate dehydrogenase (LDH) activity of H9c2 cells after different treatment. **m** Quantitative results of intracellular malondialdehyde (MDA) contents of H9c2 cells. **n**, **o** The quantification of glutathione (GSH) levels and GSH/GSSG ratio in H9c2 cells. **p** Quantitative results of intracellular ferrous iron. **q**, **r** Representative images and quantitative analysis of Western blotting analysis of GPX4, ACSL4, YAP and p-YAP in H9c2 cells after different treatment. Values represent the mean ± SD. *P < 0.05, **P < 0.01 vs. CON group. ns indicates no significance, ^#^*P* < 0.05, ^##^*P* < 0.01 vs. DOX group. ^†^*P* < 0.05, ^††^*P* < 0.01 vs. DOX + rHMGB1 group

Therefore, we aimed to validate this process in vitro. Based on the results of the CCK-8 assay, H9c2 cells were stimulated with 1 μm DOX for 24 h to establish a DIC cell injury model, while incubation with 5 μm erastin was performed for the same duration as a positive control (Fig. S7a, b). Meanwhile, the Fer-1 was employed to validate whether the DOX-induced injury in H9c2 cells was attributed to ferroptosis. The results showed that after 24 h of DOX stimulation, cell viability in the DOX and erastin groups was significantly lower than that in the CON group (Fig. 7a). Additionally, there was an increase in LDH levels. However, treatment with Fer-1 effectively ameliorated these changes (Fig. 7b). Furthermore, similar to those in the erastin group, MDA levels were increased, while GSH levels and the GSH/GSSG ratio were significantly reduced in the DOX group, but administration of Fer-1 resulted in evident remission (Fig. 7c–e). Iron metabolism dysregulation is a distinctive characteristic of ferroptosis. The analysis of ferrous ions revealed a significant increase in the DOX group and erastin group, and there was a notable decrease in the DOX + Fer-1 group compared to the DOX group (Fig. 7f). Moreover, we validated the changes in various proteins at the cellular level by Western blot analysis. The results showed a decrease in GPX4 protein expression and an increase in ACSL4 protein expression in the DOX group, which was consistent with the changes observed following erastin stimulation. Notably, treatment with Fer-1 mitigated these alterations (Fig. 7g, h). These findings provide further confirmation of ferroptosis in DOX-induced cardiomyocytes. The expression levels of YAP and phospho-YAP were consistent with those observed in vivo.

To further investigate the involvement of HMGB1 in DOX-induced ferroptosis and its downstream targets, rHMGB1 and the specific TLR4 inhibitor TAK242 were incubated with cells, and cellular damage and ferroptosis indicators were assessed. Based on the cck-8 results, 1 ng HMGB1 were selected and co-incubated with 1 μm DOX in H9c2 cells (Fig. S7c). Initially, we observed the alterations in TLR4 expression and evaluated the inhibitory efficacy of TAK242 among different groups using western blot (Fig. 7i, j). After verifying the effective inhibition of TLR4, subsequent experiments were carried out. First, the CCK-8 results showed a significantly reduction in cell viability in the DOX + rHMGB1 group than in the DOX group. Conversely, treatment with TAK242 slightly increased cell viability (Fig. 7k). Additionally, DOX and rHMGB1 increased cellular LDH levels, which were subsequently reduced by TAK242 treatment (Fig. 7l). These findings suggest that further stimulation with rHMGB1 exacerbates DOX-induced cellular damage, while inhibiting TLR4 mitigates the damage caused by DOX and rHMGB1. Compared with the DOX group, the DOX + rHMGB1 group exhibited increased levels of MDA and ferrous ions, reduced levels of GSH and a decreased GSH/GSSG ratio (Fig. 7m–p). However, inhibition of TLR4 ameliorated these changes associated with ferroptosis. Finally, of the TLR4 inhibitor abrogated the decrease in YAP and increase in p-YAP caused by DOX and rHMGB1 and reversed the decreases in GPX4 and increase in ACSL4 expression, which are related to ferroptosis. This finding provides evidence that rHMGB1 affects YAP expression through TLR4 and regulates ferroptosis to aggravate damage caused by DOX. Furthermore, the changes in protein levels provide an intuitive view. Results demonstrated that the inhibition of TLR4 effectively reversed the downregulation of YAP and upregulation of p-YAP induced by DOX and rHMGB1, the same reversal effect were also observed in the proteins GPX4 and ACSL4 (Fig. 7q, r). The present findings suggest that rHMGB1 regulates the expression of YAP through TLR4 and modulates ferroptosis to exacerbate DOX-induced damage.

## Discussion

The pathogenesis of DIC is intricate and involves multiple mechanisms and interactions [22]. Our previous study revealed the involvement of the Hippo pathway in DOX-induced ferroptosis in cardiomyocytes [11]. In this study, we established an animal model of DIC through periodic administration of DOX, and we confirmed the occurrence of ferroptosis in DIC and identified an inflammatory response following DOX treatment that was characterized by the infiltration of neutrophils. Subsequently, NETs were examined, and neutrophils were depleted using neutrophil-neutralizing antibodies targeting Ly6G. Notably, neutrophil depletion ameliorated DOX-induced myocardial damage. The occurrence of NETs is concomitant with the extracellular release of substantial amounts of HMGB1, which alters the immune microenvironment of myocardial tissue. We further validated that inhibition of NETs-mediated HMGB1 could modulate the activation of the Hippo pathway via TLR4 in vivo, thereby mitigating ferroptosis in cardiomyocytes. This study is the first investigation of the impact of NETs on ferroptosis induced by DOX treatment and shed light on the mechanism of DIC by focusing on alterations in the immune microenvironment.

Although the cardiotoxicity of DOX has long been recognized, DOX remains widely used as a chemotherapy agent, particularly in the treatment of pediatric malignancies. Statistically, approximately 50% of pediatric malignant tumors are managed through the administration of DOX [23, 24]. Currently, there is a paucity of effective clinical strategies available for treating DOX-induced myocardial damage. The prevention of DIC has been greatly restricted because of the limited ability to accurately predict which patients will develop cardiotoxicity and its severity. Furthermore, reducing the dosage to prevent adverse effects may compromise the efficacy of primary malignancy treatment. The availability of clinically approved drugs for the treatment of DIC is severely limited, and the effectiveness of drugs other than dexrazoxane remains uncertain [25]. Therefore, further investigations of additional mechanisms underlying DIC and the identification of effective targets to mitigate this injury are crucial.

In addition to the initially proposed mechanism of oxidative stress, various processes, including inflammation, autophagy, apoptosis, and ferroptosis, are involved in the pathogenesis of DIC [26–29]. The form of programmed cell death known as ferroptosis is characterized by dysregulated iron ion metabolism, the accumulation of ROS, and lipid peroxidation [30]. This phenomenon has gained significant attention in recent years. Numerous studies have demonstrated the role of ferroptosis in DIC. Fang X et al. reported that the survival rates of mice treated with inhibitors of apoptosis, necrosis, and autophagy were lower than those of mice treated with a ferroptosis inhibitor after DOX administration. Furthermore, they demonstrated that the administration of an iron chelating agent significantly mitigated myocardial toxicity induced by DOX [29]. The mechanism by which DOX induces ferroptosis in cardiomyocytes is intricate and governed by diverse signaling pathways [22]. Our previous study revealed the involvement of the Hippo pathway in DOX-induced cardiomyocyte ferroptosis.

The Hippo pathway is a regulatory pathway that controls the expression of target genes through the phosphorylation of downstream transcriptional coactivators, which is facilitated by a cascade of serine/threonine kinases. YAP is a pivotal effector molecule in the Hippo pathway that regulates tissue homeostasis, development, and tumor progression. Many studies have elucidated the role of the Hippo pathway in ferroptosis [31, 32]. It has been shown that the phosphorylation of YAP at Ser127 induces its binding to 14-3-3, resulting in its inability to enter the nucleus [33]. This process is controlled by a multitude of upstream regulatory factors, including cell polarity, adhesion proteins, mechanical transduction, and various signaling pathways [34, 35]. Considering the sensitivity of the Hippo pathway to extracellular signals, we hypothesized that alterations in YAP during DOX-induced ferroptosis were potentially influenced by changes in the extracellular environment. Several studies have shown that YAP signaling may also be activated by inflammatory response [68]. There are also scholars have shown more specifically that the delay in diabetic wound healing resulting from endothelial-mesenchymal transition was induced by regulation of the Hippo pathway by NETs [37]. These findings provides further supports for our hypothesis. Moreover, Zhang S. et al. observed a significant increase in inflammatory cytokines in the peripheral blood following DOX treatment in mice [38]. Hu et al. demonstrated that DOX-induced myocardial inflammation was ameliorated in vivo and in vitro by the administration of Osteocrin, thereby mitigating DIC [39]. These findings suggested that changes in the immune microenvironment induced by the inflammatory response and innate immunity may play important roles in DIC. We also confirmed the occurrence of myocardial tissue inflammation following DOX stimulation by assessing the levels of the cytokines TNF-α, IL-6, IL-8, and CXCL1 and by performing HE staining of myocardial tissue.

Neutrophils, which are the most abundant immune cells in the body, are the primary inflammatory cells that initiate an immune response [15]. In response to stimulation by exogenous pathogens, the body induces a rapid increase in neutrophils through the production of chemokines such as interleukins. This response plays a protective role by facilitating adhesion, phagocytosis, or degranulation to prevent the dissemination of pathogens and subsequent systemic infection [40]. In the context of an aseptic inflammatory response, neutrophils play a crucial role in the efficient clearance of cellular debris at the site of injury [41]. Moreover, in addition to their own phagocytic activity, neutrophils can also promote macrophage infiltration, facilitating the rapid elimination of necrotic cells [42]. Neutrophils can amplify the inflammatory response. Although the primary purpose of these cells is to facilitate the rapid response to pathogen invasion, the outcomes of this behavior may not always be favorable, particularly in cases of aseptic inflammation [43, 44]. NETs have been identified as an additional defense mechanism of neutrophils [16]. NETs formation occurs when activated neutrophils release depolymerized DNA outside the cell to form a network structure and simultaneously release nuclear proteins such as histones/HMGB1. The reticular structure exhibits adhesive properties that enable it to ensnare pathogens while simultaneously releasing bioactive molecules that enhance the inflammatory response and facilitate the transduction of various cellular signals [45]. The pivotal role of NETs in the pathogenesis of various diseases, including malignant tumors, myocardial infarction, and myocarditis, has been extensively demonstrated by numerous studies [46]. Todorova et al. performed a clinical observational study and showed that the levels of circulating NETs markers, such as serum CRP, MPO, and dsDNA, were increased in patients experiencing changes in LVEF following DOX chemotherapy. The authors suggested that these indicators could serve as significant predictors of DIC [19]. This discovery prompted us to consider that, in addition to serving as a predictor, NETs are highly likely to play a vital role in the pathogenesis of DIC. In this study, we showed the occurrence of NETs in DIC in vivo and that depleting neutrophiles with a neutralizing antibody effectively mitigated the production of NETs, thereby leading to a reduction in myocardial damage.

However, the underlying mechanism by which NETs contribute to myocardial impairment in DIC remains elusive. We hypothesize that the release of cellular contents during NETs formation induces alterations in the immune microenvironment surrounding cardiomyocytes. These alteration may potentially be linked to the activation of the Hippo-YAP signaling pathway. Among factors released by NETs, HMGB1 has captured our attention. The primary biological function of HMGB1 is to participate in the maintenance of nuclear structure [47]. When it is released extracellularly, it plays a crucial role as a late-stage inflammatory mediator. HMGB1 plays a role in the pathogenesis of infectious diseases, such as sepsis and myocarditis [48, 49]. However, its functionality extends beyond that studies have revealed the involvement of HMGB1 in various biological processes, such as endothelial injury, oxidative stress, and pyroptosis even NETs [50, 51]. There are also scholars conducted investigations into the involvement of HMGB1 in the process of DIC. Luo P et al. reported that HMGB1 modulated DIC by upregulating autophagy [52]. Lv et al. have demonstrated that the attenuation of intracellular HMGB1 downregulated autophagy via Akt/mTOR signaling pathway, thereby mitigating cardiac damage in DIC [53]. Alzokaky et al. demonstrated that HMGB1 levels are elevated in myocardial tissue following DOX treatment and suggested its involvement in myocardial injury through mechanisms such as inflammation and oxidative stress [54]. Zhang et al. showed the increase of HMGB1 in myocardial tissue after DOX stimulation in rats and demonstrated that DXZ alleviated ferroptosis and cardiomyopathy through regulating HMGB1 [55]. However, the investigation into the origin of increased HMGB1 was not undertaken in these studies. Our study not only confirmed a significant increase in HMGB1 expression in the myocardial tissue of mice following DOX stimulation. Furthermore, we also observed that inhibiting the occurrence of NETs reduced HMGB1 levels. It is suggesting that a substantial portion of extracellular HMGB1 originated from NETs generation. It provides insights into the underlying mechanisms leading to HMGB1 elevation prior to cellular demise and reinforce the groundwork for investigating the early involvement of HMGB1 in DIC. On the one hand, an excessive amount of extracellular HMGB1 can potentiate inflammatory responses, which is a deleterious process. Maugeri N et al. demonstrated that HMGB1 facilitated the generation of NETs [56]. This implies that the release of HMGB 1 by NETs may establish a positive feedback loop, thereby facilitating the exacerbation of NETs. The verification of this statement should be conducted in subsequent experiments. On the other hand, HMGB1 may also play a regulatory role in modulating other cellular damage processes. For instance, Wang et al. demonstrated the involvement of HMGB1 in ferroptosis during acute liver failure [57]. The regulatory role of HMGB1 in high glucose-induced ferroptosis in mesangial cells was also confirmed by Wu et al. [58]. Moreover, our study also revealed a strong correlation between an increase in HMGB1 levels and reduced YAP expression or ferroptosis induced by DOX in cardiomyocytes, and these changes were rescued by inhibiting NETs and decreasing HMGB1 levels. Furthermore, the restoration of YAP expression and changes in ferroptosis were observed after NETs inhibition combined with the administration of rHMGB1. These findings suggest that NETs aggravated cardiomyocyte ferroptosis via the Hippo pathway through HMGB1 release.

In the context of cellular stress or tissue injury, extracellular HMGB1 can interact with various cell receptors and play distinct roles in facilitating inflammatory responses and promoting tissue regeneration [59, 60]. However, if this signaling pathway is significant or prolonged, it can initiate the progression of other cellular damage mechanisms and subsequently lead to tissue damage. HMGB1 typically interacts with receptors such as RAGE/TLR4/TLR2, which are located on myocardial cell membranes [61–63]. We used RT‒qPCR to assess the levels of these receptors in mouse myocardial tissue before and after DOX stimulation and NETs inhibition. Our findings revealed an increase in TLR4 mRNA level in this animal model, which was further supported by the immunoblot results, demonstrating the impact of NETs regulation on TLR4 expression. Relevant investigations of the role of the HMGB1/TLR4 axis in cardiovascular diseases are not uncommon. Ding et al. revealed the involvement of the HMGB1/TLR4 axis in the regulation of apoptosis during ischemia‒reperfusion injury [64]. Qi et al. also shown in their study, that the regulation of HMGB1 translocation by alpha-lipoic acid was found to effectively mitigate IRI via the TLR 4/NF-κB signaling pathway [65]. Additionally, studies have demonstrated a significant correlation between the downregulation of YAP and the upregulation of TLR2 and TLR4 expression [66]. Luo et al. reported that the MyD88-dependent Toll-like receptor signaling pathway activated NF-κB and modulated the Hippo pathway during hepatitis B virus infection [67]. Zhang et al. discovered that the translocation of cytoplasmic YAP into the nucleus, which was mediated by HMGB1-TLR2, could further induce the dedifferentiation of CD133^−^ cancer cells [36]. Consideration was given to the activation of the Hippo pathway in DOX-induced ferroptosis in cardiomyocytes, which resulted from excessive binding between HMGB1 and TLR4. Therefore, we performed in vitro experiments, and H9c2 cells were stimulated with DOX and an excess of HMGB1. After the application of TLR4-specific inhibitors, cell viability was significantly increased, and this change was accompanied by reduced YAP phosphorylation and the attenuation of ferroptosis. These findings provided additional evidence to support our hypothesis. Our findings substantiate the crucial role of HMGB1 induced by NETs in ferroptosis-mediated DIC and highlight the regulatory association between HMGB1/TLR4 and YAP. However, further investigations are still needed to determine the intracellular pathway by which TLR4 activation regulates YAP phosphorylation.

Limitations of this study: First, this study exclusively focused on the association between NETs and ferroptosis due to the recognition of ferroptosis as the primary mode of cell death in DIC. However, alternative programmed cell death mechanisms, such as pyronecrosis and apoptosis, exist during DIC, necessitating further experimental investigations to determine the interplay between NETs and these distinct modes of programmed cell death. Second, the occurrence of NETs is accompanied by the release of other components, such as MPO and NE; however, the impact of these other factors on myocardial injury was not explored in this study.

In conclusion, our findings suggest that DOX-induced NETs production modulates ferroptosis in cardiomyocytes via the HMGB1/TLR4/YAP axis, thereby contributing to myocardial injury. These results offer a novel approach for preventing and alleviating DIC by targeting alterations in the immune microenvironment.

### Supplementary Information

Below is the link to the electronic supplementary material.

Supplementary file1 (PDF 907 KB)

## Data Availability

All data generated or analysed during this study are included in this published article and the supplementary data. Data will be made available on request.
