# Supplementary material for: Neutrophil extracellular traps mediate cardiomyocyte ferroptosis via the Hippo–Yap pathway to exacerbate doxorubicin-induced cardiotoxicity
Source: Cell Mol Life Sci. 2024 Mar 8;81(1):122. doi: 10.1007/s00018-024-05169-4 (PMC10923748; doi:10.1007/s00018-024-05169-4)
Supplement: Supplementary file 1 — Supplementary file1 (PDF 907 KB) [file 18_2024_5169_MOESM1_ESM.pdf]

Neutrophil extracellular traps mediate cardiomyocyte ferroptosis via the Hippo–Yap pathway to exacerbate doxorubicin-induced cardiotoxicity

Cellular and Molecular Life Sciences

Peng Zhao[a,c,#], You Li[a,c,#], Xiangli Xu[b], Haobo Yang[a,d], Xintong Li[a,d], Shuai Fu[a,c], Zihong Guo[a], Jianing Zhang[a], Hairu Li[a,*], Jiawei Tian[a,*]

* Corresponding authors:

Postal address: Department of Ultrasound , the Second Affiliated Hospital of Harbin Medical University, Harbin 150001, China

Tel./Fax: +86 0451 86605253(Jiawei Tian) and +86 0451 86605811 (Hairu Li)

E-mail address: jwtian2004@163.com (Jiawei Tian) and lihairu0419@126.com (Hairu Li).

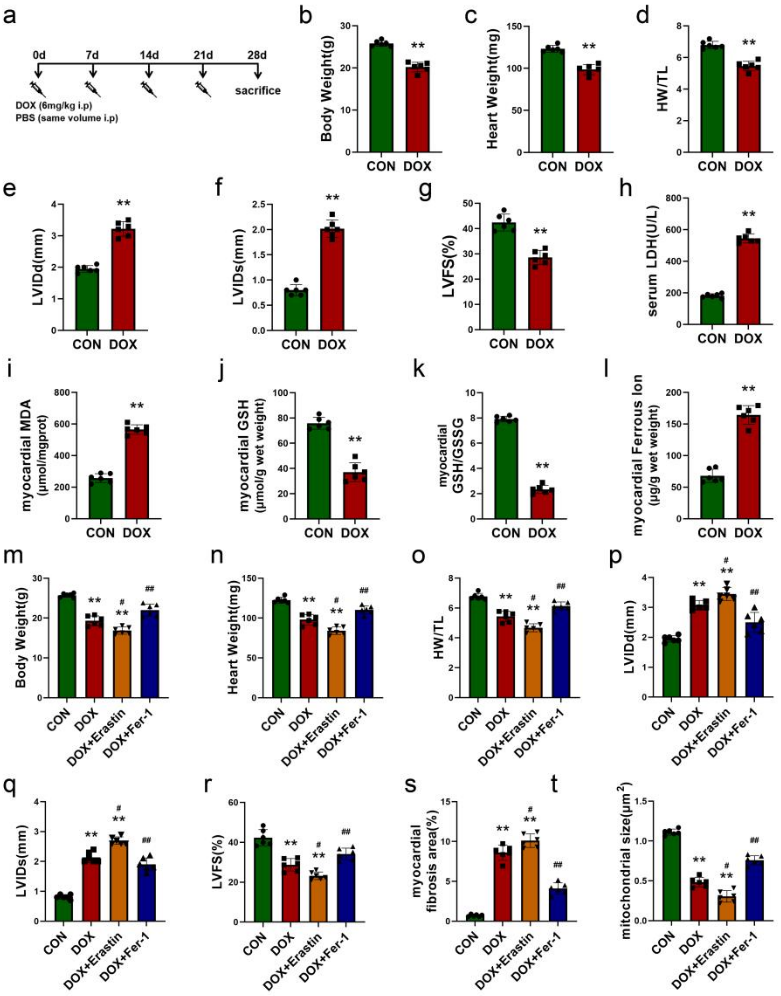

Supplementary Fig. 1 Ferroptosis plays a pivotal role in the pathogenesis of cardiac damage associated with DIC

a Experimental protocol. **b** Body weight (after 28 days of DOX treatment, the same applies to C-L). **c** Heart weight. **d** Ratio of heart weight to tibial length (HW/TL). **e, f** Quantifications of left ventricular internal diameter in systole and diastole (LVIDs and LVIDd). **g** Quantifications of left ventricular fraction shortening (LVFS). **h** The levels of lactate dehydrogenase (LDH). **i** Quantitative results of myocardial malondialdehyde (MDA) contents. **j, k** Quantitative results of myocardial glutathione (GSH) and GSH/GSSG. **l** Quantifications of the ferrous iron levels in myocardium. **m** Body weight (after DOX, Erastin and Fer-1 treatment, the same applies to N-T). **n, o** Heart weight and ratio of heart weight to tibial length (HW/TL). **p, q** Left ventricular internal diameter in systole and diastole (LVIDs and LVIDd) of mice in each group. **r** Quantifications of left ventricular fraction shortening (LVFS). **s**

Quantitative results of sirus red staining. **t** Quantitative results of mitochondrial area obtained through transmission electron microscope. Values represent the mean±SD. *P < 0.05, **P < 0.01 vs. CON group. #P < 0.05, ##P < 0.01 vs. DOX group.

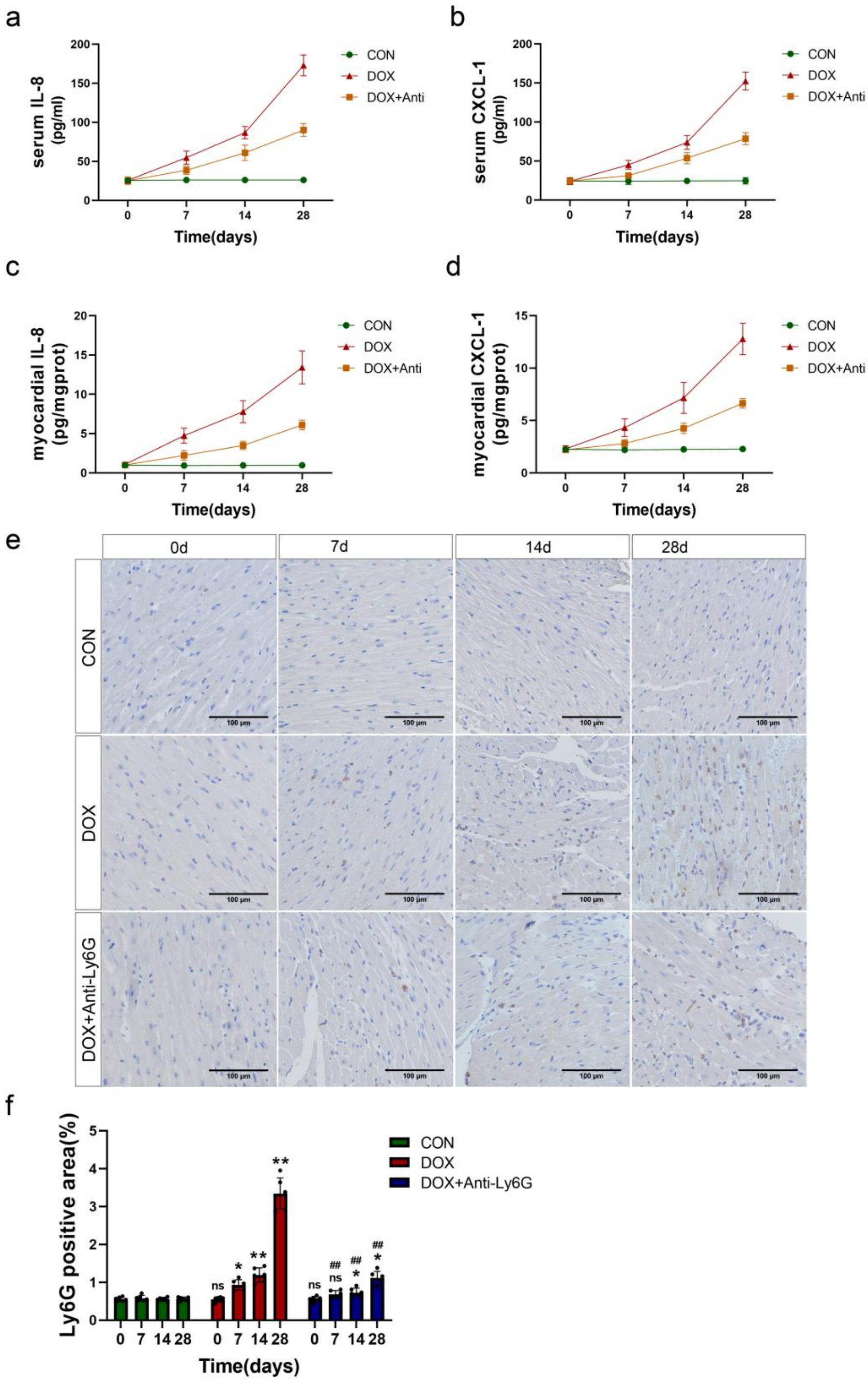

Supplementary Fig. 2 Neutrophil infiltration at different time points following administration of DOX and conducted combined depletion of neutrophils

a, b, c, d The levels of cytokines IL-8 and CXCL-1 in serum and myocardial content after DOX and neutrophil depletion treatment at different times. **e, f** Representative images and quantitative results of Ly6G immunohistochemical staining were obtained at different time points following DOX and neutrophil depletion treatment (scale bar, 200μm). Values represent the mean±SD. ns indicates no significance, *P < 0.05, **P < 0.01 vs. CON group at the same time point. $^{\#}P$ < 0.05, $^{\#\#}P$< 0.01 vs. DOX group at the same time point.

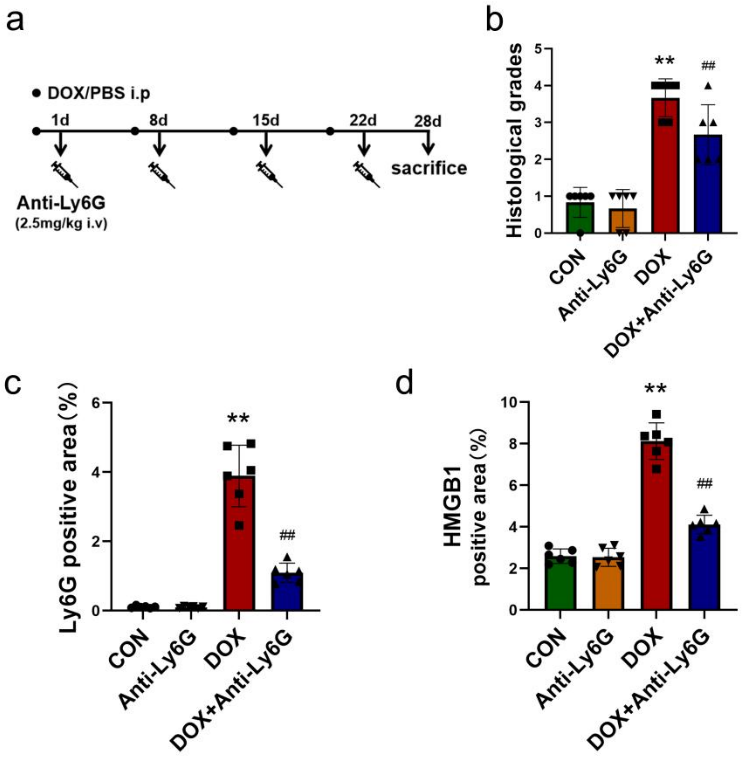

Supplementary Fig. 3 Neutrophil depletion of DIC mice attenuates the production of NETs and the release of HMGB1

a Experimental protocol. **b** Quantitative results of H&E staining. **c** Quantitative results of Ly6G immunohistochemical staining. **d** Quantitative results of HMGB1 immunohistochemical staining. Values represent the mean±SD. *P < 0.05, **P < 0.01 vs. CON group. $^{\#}P$ < 0.05, $^{\#\#}P$ < 0.01 vs. DOX group.

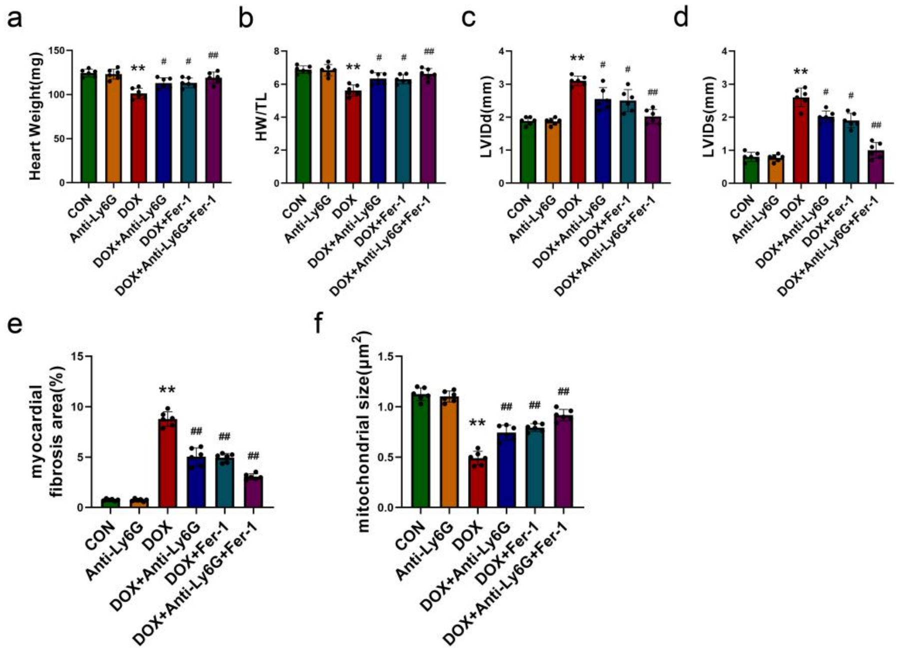

Supplementary Fig. 4 Inhibition of NETs alleviates doxorubicin-induced myocardial injury by reducing ferroptosis

a, b Heart weight and ratio of heart weight to tibial length (HW/TL). **c, d** Left ventricular internal diameter in systole and diastole (LVIDs and LVIDd) of mice in each group. **e** Quantification of left ventricular fraction shortening (LVFS). **f** Quantitative results of sirus red staining. **g** Quantitative results of mitochondrial area obtained through transmission electron microscope. Values represent the mean±SD. *$P < 0.05$, **$P < 0.01$ vs. CON group. $^{\#}P < 0.05$, $^{\#\#}P < 0.01$ vs. DOX group.

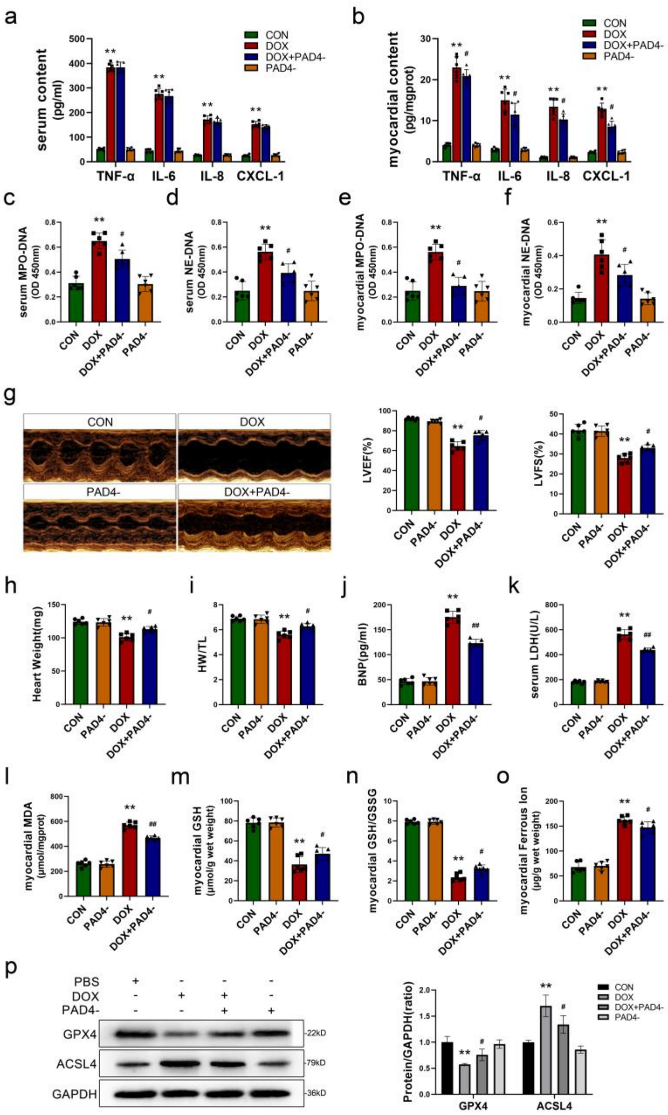

Supplementary Fig. 5 Inhibition of NETs ameliorates doxorubicin-induced myocardial injury and attenuates ferroptosis

a, b The content of cytokines TNF-α, IL-6, CXCL-1 and IL-8 in serum and myocardial tissues after DOX and PAD4 inhibitor treated. **c, d, e, f** Quantitative analysis of serum and myocardial tissues MPO-DNA and NE-DNA complexes by ELISA kits. **g** Representative images of M-mode echocardiography and quantification of left ventricular ejection fraction (LVEF) and left ventricular fraction shortening (LVFS). **h, i** Heart weight and ratio of heart weight to tibial length. **j, k** The brain natriuretic peptide (BNP) and lactate dehydrogenase (LDH) levels in serum. **l** Quantitative results of malondialdehyde (MDA) levels in myocardial tissues. **m, n** Quantitative results of myocardial

glutathione (GSH) and GSH/GSSG ratio. **o** The quantifications of ferrous iron in myocardium. **p** Representative images and quantitative analysis of Western blotting analysis of GPX4 and ACSL4. Values represent the mean±SD. *$P < 0.05$, **$P < 0.01$ *vs.* CON group. $^{\#}P < 0.05$, $^{\#\#}P < 0.01$ ***vs.*** DOX group.

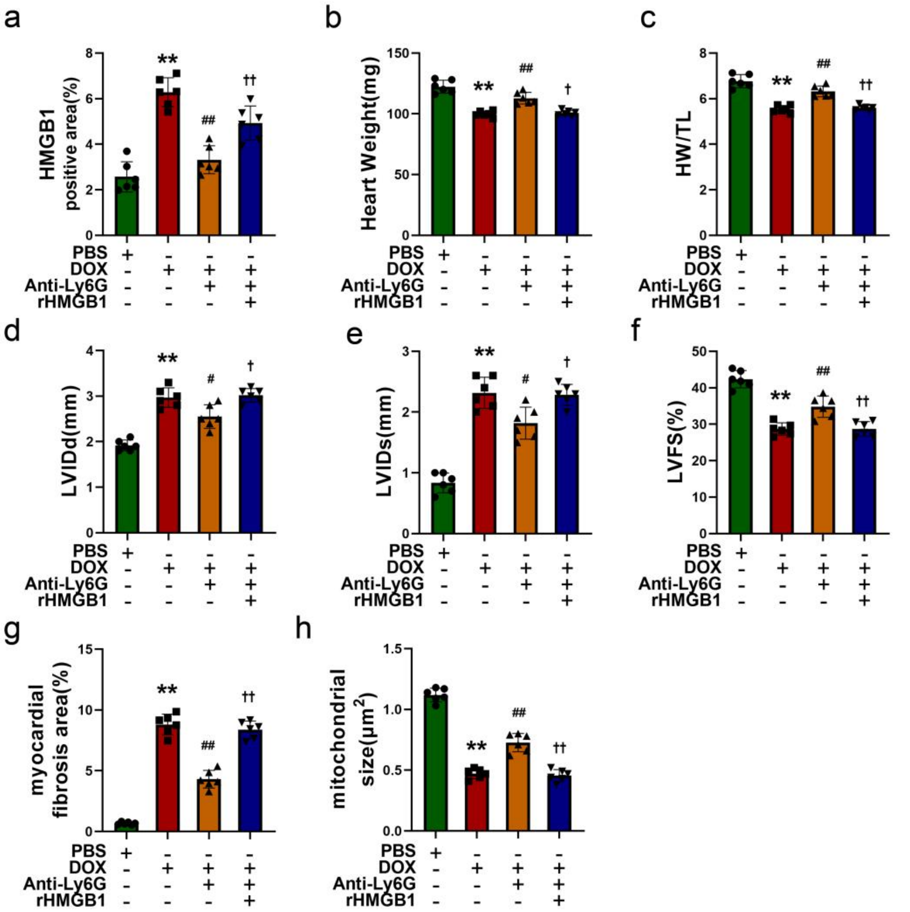

Supplementary Fig. 6 HMGB1 serve as a crucial regulatory factor in the reduction of ferroptosis mediated by NETs inhibition in DIC

a Quantitative results of HMGB1 immunohistochemical staining after rHMGB1 injected. **b, c** Heart weight and ratio of heart weight to tibial length (HW/TL) in each group. **d, e** Left ventricular internal diameter in systole and diastole (LVIDs and LVIDd) of mice in each group. **f** Quantification of left ventricular fraction shortening (LVFS). **g** Quantitative results of sirus red staining. **h** Quantitative results of mitochondrial area obtained through transmission electron microscope. Values represent the

mean±SD. *$P < 0.05$, **$P < 0.01$ vs. CON group. $^{\#}P < 0.05$, $^{\#\#}P < 0.01$ vs. DOX group. $^{\dagger}P < 0.05$, $^{\dagger\dagger}P < 0.01$ vs. DOX+Anti-Ly6G group.

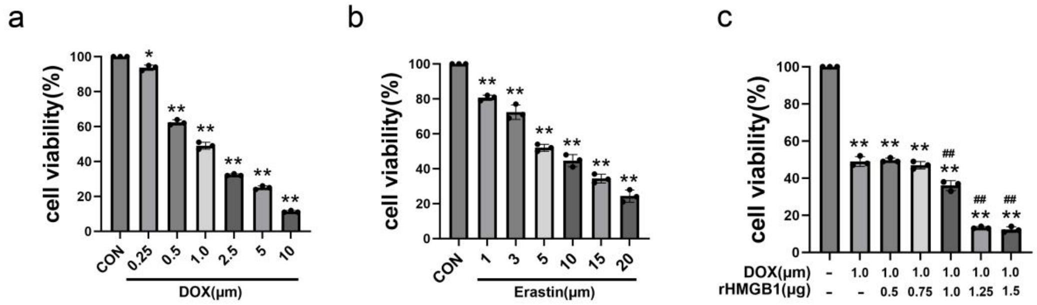

Supplementary Fig. 7 Results of the CCK-8 assay following exposed to different reagent concentrations

a H9c2 cells exposed to different concentrations of DOX for 24h. **b** H9c2 cells incubated with different concentrations of erastin for 24h. **c** H9c2 cells incubated with 1 μm DOX and different concentrations of rHMGB1 for 24h. Values represent the mean±SD. *$P < 0.05$, **$P < 0.01$ vs. CON group. $^{\#\#}P < 0.01$ vs. DOX group.
